# Spatially explicit paleogenomic simulations support cohabitation with limited admixture between Bronze Age Central European populations

**DOI:** 10.1038/s42003-021-02670-5

**Published:** 2021-10-07

**Authors:** Jérémy Rio, Claudio S. Quilodrán, Mathias Currat

**Affiliations:** 1 Department of Genetics and Evolution - Anthropology Unit, University of Geneva, Geneva, Switzerland; 2 Department of Zoology, University of Oxford, Oxford, United Kingdom; 3 Institute of Genetics and Genomics in Geneva (IGE3), University of Geneva, Geneva, Switzerland

**Keywords:** Population genetics, Anthropology

## Abstract

The Bronze Age is a complex period of social, cultural and economic changes. Recent paleogenomic studies have documented a large and rapid genetic change in early Bronze Age populations from Central Europe. However, the detailed demographic and genetic processes involved in this change are still debated. Here we have used spatially explicit simulations of genomic components to better characterize the demographic and migratory conditions that may have led to this change. We investigated various scenarios representing the expansion of pastoralists from the Pontic steppe, potentially linked to the Yamnaya cultural complex, and their interactions with local populations in Central Europe, considering various eco-evolutionary factors, such as population admixture, competition and long-distance dispersal. Our results do not support direct competition but rather the cohabitation of pastoralists and farmers in Central Europe, with limited gene flow between populations. They also suggest occasional long-distance migrations accompanying the expansion of pastoralists and a demographic decline in both populations following their initial contact. These results link recent archaeological and paleogenomic observations and move further the debate of genomic changes during the early Bronze Age.

## Abstract (web summary)

Rio et al. design and utilise spatially explicit simulations of genomic components to investigate the cohabitation or competition hypothesis of Bronze Age Central European human populations. Their results support cohabitation with limited gene flow between pastoralists and farmers, linking together palaeogenomic and archaeological observations from the literature.

## Introduction

While the rise of the farming lifestyle during the Neolithic transition has attracted the attention of population geneticists for decades (e.g.,^1–4^), little is known about the population interactions and dynamics during the period marking the rise of the Bronze Age (BA) in Central Europe (CE). The recent developments allowing DNA sequencing from ancient individuals represent a promising method for better understanding this period of complex social, cultural, and economic changes (e.g.,^5^), representing one of the key stages of human prehistory in western Eurasia.

Recent paleogenomic studies focusing on the British islands and the Iberian Peninsula showed major changes in the human gene pool during the BA. The British island populations experienced a nearly total replacement of the local population at approximately 4400 years Before Present (BP)^6^, while southwestern Europe experienced a total replacement of male lineages at about the same time (4500–4000 BP^6,7^). These studies indicated that genetic components related to the populations of the Pontic steppe (hereafter called steppe ancestry) were introduced during this period. While it is widely accepted that people of steppe ancestry are involved in these genetic replacement events^6–8^, it is less clear the extent to which they are related to the Yamnaya cultural complex (YCC, also called Pit-Grave culture) that originated in the Pontic steppe at approximately 6000–5300 BP^9,10^. Despite the wide range of cultures identified in the archeological record that characterize this period, the YCC is central to the debate due to the spread of its funeral ritual^11^ and associated genetic background^12–14^ over large areas.

There is around 3500 *km* between the Atlantic fringes of Europe and the Pontic steppe, with CE being key for studying the connection between these two areas during the BA. Populations from CE have been shown to hold a large amount of steppe ancestry that appeared within a short period of time. “Funnelbeaker”, “Baden” and “Globular Amphora” are diverse CE cultures associated with the fifth millennium BP. They predate the Corded Ware cultural complex (CWC), one of the earliest archeological culture to have been genetically associated with steppe ancestry^12,15^. While the origin of the CWC has been questioned in the past^9^, its connection to the YCC has been strengthened recently^12,16–18^ but is still debated^13,19–21^. On the one hand, the archeological record supports an impact of the YCC on Central European cultures based on mixed assemblages (i.e., sex distinctions in burial traditions)^18^. There are similarities in burial rites, but a distinction can be made based on differences in potteries^17,18^. On the other hand, recent paleogenomic analyses support a large amount of steppe ancestry in CWC populations^13,21–24^. Thus far, arguments from both the archeology and paleogenomics point to a close relationship between the populations associated with two geographically distant cultural complexes, with the YCC starting earlier but partially overlapping spatially and temporally with the CWC^17^.

Haak et al.^12^ interpreted the rapid and large genomic shift in CE during the BA as the consequence of massive migrations from the steppes. The term “massive” does not necessarily mean a high number of individuals and could actually imply a relatively small size of local populations in comparison to the migrating populations from the steppe. However, little is known about the relative population sizes of various communities during the BA, contrary to the Neolithic transition, during which hunter-gatherer populations were replaced by farmer populations able to sustain much larger densities^25–27^. Furthermore, the massive migrations hypothesis is contradicted on the archeological grounds^3^, which shows no sign of massive migrations but rather a more diffused process through a deeper, long-term history of interactions characterized by regular and repeated movements between the Pontic steppe and CE. While there is no doubt that some migration of YCC-related populations occurred from the Pontic steppes westward around 5000 BP, more work needs to be done to understand the demographic processes that have shaped the genetic diversity of human populations during the BA^11^.

Interestingly, the period associated with the YCC and CWC encompasses the time of the most ancient detected traces of *Yersinia pestis*, the pathogen responsible for the plague. Rasmussen et al.^28^ proposed that strains of *Y. pestis* from the BA did not carry the *Yersinia* murine toxin gene (or YMT gene), which gives rise to flea-borne transmission of the plague and the ability to cause bubonic plague, but Demeure et al.^29^ proposed that this gene was acquired by a different branch of *Y. pestis* that split around 5300 BP. One question that emerged is whether *Y. pestis* would have played a role during the diffusion of pastoralists from the Pontic steppe to CE during the BA^28,30,31^. It could indeed be hypothesized that incoming pastoralists arrived with pathogens to which they were resistant but Central Europeans were not (or less so)^28^. These pathogens may then have decimated part of the indigenous populations, allowing the genomes of incoming pastoralists to partially replace local genomes without a large number of migrants.

The population dynamics and interactions of populations during the BA in CE remain unclear. It has been shown that eco-evolutionary simulations of paleogenomic data could provide insights into these processes^32^. Spatially explicit simulations applied to human evolution have been developed to study the European Neolithic transition. They have provided important information, which could not have been identified otherwise, on the genetic consequences of the interactions between early farmers and hunter-gatherers^1,33,34^, including the effects of sex-biased migrations and variable admixture rates^5^. Although focusing on the Neolithic transition, a couple of studies^33,34^ also incorporated migrations from the steppes in their simulations that were applied to allele frequencies. The spatially explicit approach implemented in the program SPLATCHE3^35^ offers an adequate tool for addressing further questions related to these topics as it allows the simulation of two layers of interacting populations that admix at various levels over time and space (e.g.,^5^). In the BA context, one layer would represent the Central European populations characterized by sedentary farmers, while the second layer would represent the incoming populations from the Pontic steppe, characterized by herding and the use of horses. The farmer and pastoralist layers would thus represent structured populations having different ways of life and possibly other distinct characteristics (density, growth, and migration rates). SPLATCHE3 can generate virtual population samples with the same sizes, locations, and dates as the real samples, from which the proportions of alleles coming from one or another population source (here, pastoralists or farmers) can be computed for multiple loci (e.g.,^36^).

**Fig. 1:**
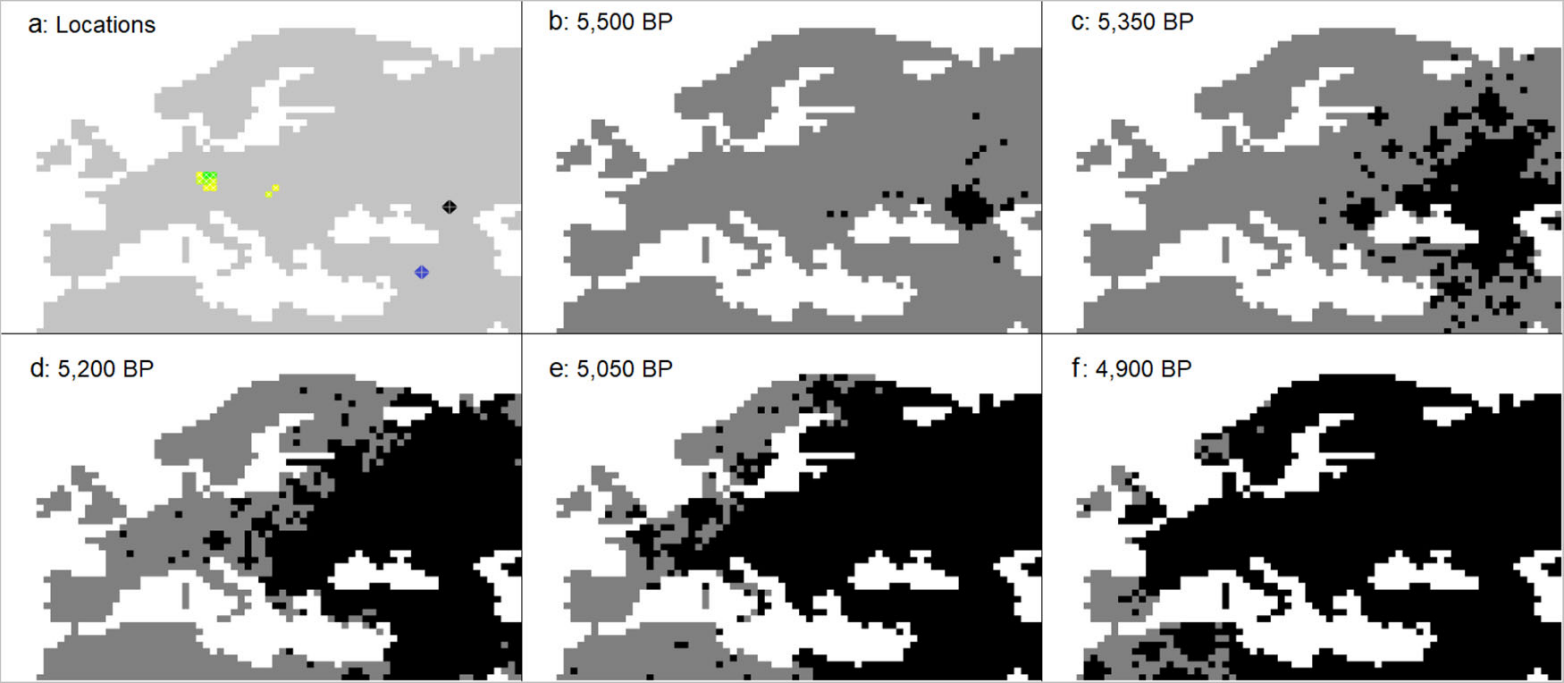
Illustration of the simulation map. Each colored cell represents 100 by 100 *km* of land. The white cells represent water. **a**) Simulated sampling locations; light to dark green represents 1–10 diploid paleogenomes per deme (see Table 1), the blue dot shows the chosen source for the farmer layer, and the black dot shows the chosen source for the pastoralist layer. **b**–**f**) Example of the simulation of pastoralist dispersal short- and long-distance migrations. The dark gray cells contain populations that precede the pastoralists, whether they were farmers or hunter-gatherers, and the black cells also contain the pastoralists.

In our study, we used complex spatially explicit simulations (Fig. 1) combined with a Bayesian estimation procedure to compare virtual genomic proportions simulated under various scenarios of the expansion of pastoralists from the Pontic steppes towards CE during the BA, with the genomic ancestry components estimated with 34 paleogenomes from 10 populations dating from this period^12^. We aim to clarify the conditions under which contacts between the local farmers and incoming pastoralists during the BA resulted in the sudden appearance of large steppe ancestry components in CE^12,13^. We explored various scenarios of the expansion of pastoralist populations from the Pontic steppe, where the YCC originates, westward and their admixture with the local farming populations of CE. Our scenarios include i) symmetrical gene flow between populations, ii) competition within and between populations, iii) short- and long-distance dispersal, iv) various effective population sizes, and v) demographic decline in one or both populations after their initial contact to represent the effects of a deadly disease, such as plague. Our goal was to reconcile paleogenomics and archeological observations.

## Results

Thirty thousand simulations with different combinations of parameters were performed for each investigated scenario. At the end of each simulation, the proportion of genes coming from either the initial pastoralist population or from the farming population was measured in each virtual sampled genome. These samples were taken at the same place and time as the observed samples^12^ (Table 1) by using a serial coalescent algorithm implemented in SPLATCHE3. An approximate Bayesian computation (ABC^37^) was used to evaluate the probability of the investigated scenario and to estimate the parameter values.

**Table 1:** Dates and locations of the samples used in the analyses.

Population sample	Observed data					SPLATCHE3 input data				
	*n* genomes	Steppe-ancestry contribution	Earliest date calBP average	Latest date calBP average	calBP average	Earliest gen.	Latest gen.	Average gen.	Location (PlateCarreeWGS84)	
Esperstedt_MN	1	0.00	5310	5036	5173	188	199	193	5,500,000	1,300,000
HungaryGamba_CA	1	0.00	4853	4756	4805	206	210	208	5,500,000	2,300,000
Corded_Ware_LN	5	0.78	4731	4255	4493	211	230	220	5,600,000	1,300,000
Karsdorf_LN	1	0.73	4514	4425	4470	219	223	221	5,500,000	1,400,000
Alberstedt_LN	2	0.56	4427	4295	4361	223	228	226	5,600,000	1,400,000
Bell_Beaker_LN	10	0.47	4305	4186	4245	228	233	230	5,700,000	1,300,000
Benzigerode_LN	3	0.57	4208	4095	4151	232	236	234	5,600,000	1,200,000
Unetice_EBA	8	0.43	4069	3926	3998	237	243	240	5,700,000	1,400,000
HungaryGamba_BA	2	0.15	3680	3582	3631	253	257	255	5,400,000	2,200,000
Halberstadt_LBA	1	0.55	3063	2971	3017	277	281	279	5,700,000	1,200,000
Total:	34									

These data are obtained from Haak et al.^12^. The earliest and latest dates (calBP) are the averages of the lower and upper dates estimated for all the individuals from the corresponding population sample. For each calBP, the average date is associated with a generation (gen.) number in SPLATCHE3, starting from 0, which corresponds to 10,000 BP, until 400, which corresponds to the present time, considering a generation time of 25 years. Latitude and longitude are given in decimal degrees for the “PlateCarree WGS84” projection.

We tested three scenarios to simulate the effect of a disease reducing the population size of the farmers (scenario *“F”*), the pastoralists (scenario *“P”*) or both (scenario “*F*&*P*”) at the time when pastoralists arrive in CE. These three scenarios are compared to a fourth “*control*” scenario, where no demographic decline is simulated.

### Competition vs. cohabitation

We simulated all four scenarios with and without direct competition between farmers and pastoralists. Our assumption is that competition could be due to warfare or resource exploitation, for example. We estimated the posterior probability of the eight scenarios (Table 2). Our results show that all scenarios are able to reproduce the observed data, as the goodness of fit is always >0.05 (GOF *p values* > 0.32). The four scenarios without competition have the highest posterior probabilities, with the “*F*&*P*” scenario being the most likely (41.5%), followed by the “*F*” (19.5%), “*P*” (15.8%), and “*control*” (14.7%) scenarios. Assuming direct competition, the most likely scenario is the “*P*” scenario (7.4%), which is the only one of the direct competition scenarios with a posterior probability >1%. Pairwise Bayes factors provided in Supplementary Table S1.1 show that the support for models without competition against models with competition ranges from “strong” (26.61) to “extreme” (371.16), except against model “*P*”, which ranges from “anecdotal” (1.98) to “moderate” (5.59). The confusion matrix shows that there is 24% confidence that the “*F*&*P*” is correctly identified (Probability of Recovery, PR = 0.24, Supplementary Table S2.1). If we merge all the scenarios per model of competition, the posterior probability of a model without competition (81.9%, GOF *p value* = 0.70) is much higher than the probability of a model with direct competition (18.1%, *GOF p value* = 0.36), with a “strong” evidence for model without competition as revealed by the Bayes factor (10.22), and 83% probability of being identified correctly (PR = 0.83, Supplementary Table S2.2).

**Table 2:** Model choice results.

Optimization	Model	Scenario	Posterior probability	GOF *p* value
Variable sampling layers	Competition	*Control*	0.006	0.34
		*F*	0.001	0.55
		*P*	0.074	0.32
		*F*&*P*	0.005	0.56
	No Competition	*Control*	0.147	0.61
		*F*	0.195	0.72
		*P*	0.158	0.47
		*F*&*P*	0.415	0.69
Fixed sampling layers	No Competition	*Control*	0.124	0.86
		*F*	0.167	0.74
		*P*	0.164	0.70
		*F*&*P*	0.545	0.80

Posterior probability and goodness of fit (GOF) *p* values for the comparison of 8 scenarios (“*Control*”, “*F*”, “*P*”, and “*F*&*P*” with and without direct competition), without sampling layer optimization, and for the comparison of the 4 scenarios without competition (“*Control*”, “*F*”, “*P*”, and “*F*&*P*”), with sampling layer optimization.

### Demographic decline

We performed another model comparison using only the scenarios without competition and by fixing the layer of genetic sampling when possible (sampling in a single population layer, either farmers or pastoralists) to optimize the exploration of the parameter space (Supplementary material 3). As seen previously, all four scenarios have the ability to satisfactorily reproduce the dataset analysed (GOF *p values* > 0.70, Table 2). These results confirm the robustness of the previous analysis by highlighting that the “*F*&*P*” scenario has a higher posterior probability (54.5%) than the two other scenarios involving a single population decline (either “*P*” or “*F*”, 16.4% and 16.7%, respectively), and the less probable “*control*” scenario where there is no demographic decline (posterior probability of 12.4%). Pairwise Bayes factors provided in Supplementary Table S1.2 show a “moderate” support for “*F*&*P*” against the other three scenarios, but the differences between “*control*”, “*F,*” and “*P*” is “anecdotal”. In total, the posterior probabilities of the scenarios including a demographic decline amount to 88% against only 12% for the scenario without demographic decline. This implies that the incorporation of demographic declines in the model improves its fits to the observed paleogenomic dataset. The confusion matrix shows that 37% of the “*F*&*P*” scenario is correctly recovered (PR = 0.37, Supplementary Table S2.3), with 83% confidence that the most likely scenario is one including at least a demographic decline in one population (“*F*”, “*P*” or “*F*&*P*”, PR = 0.83). When comparing between the “*control*” and “*F*&*P*” scenarios only, the posterior probability of the “*F*&*P*” scenario (99.4%) is much higher than that of the “*control*” scenario (0.7%), with a “strong” evidence for the former as revealed by the Bayes factor (10.14), with 66.2% of “*F*&*P*” scenario being correctly recovered (PR = 0.66).

Our results strongly support a model of cohabitation without direct competition between pastoralists and farmers, with the best scenario corresponding to a case in which a demographic decline occurred, most likely affecting both populations, just after individuals with steppe ancestry arrived in CE.

### Demographic filter and posterior estimates

We estimated demographic parameter values through an ABC analysis using the most likely scenario (“*F*&*P*”) without competition.

There are four demographic parameters that are affected by the demographic filtering in the simulations (i.e., the ability to draw all the virtual samples from the same locations and times as the observed samples). These are the long-distance dispersal rate (*LDD*), the strength at which the demographic decline is applied (_*S*_*DD*), and the parameters representing the number of migrants between demes within the farmer layer (*Nm*_*F*_) and within the pastoralist layer (*Nm*_*P*_). Smaller values within the prior distributions of these four parameters are more likely to result into simulations that are compatible with all the sampling times and locations (Supplementary material 4). In the absence of LDD, simulated pastoralists do not reach sampling locations soon enough to fit the observed data, leading to “empty pastoralist demes” at these sampling times. Supplementary material 5 illustrates this, as six of the most ancient samples (out of the total ten populations) can never be sampled at their corresponding time in the pastoralist layer (in none of 10,000 simulations with LDD = 0 under scenarios “*control*” and “*F*&*P*”). This implies that LDD is necessary in our simulation framework for the expansion of pastoralists to be consistent with the dates of the appearance of their associated genetic input in CE.

**Fig. 2:**
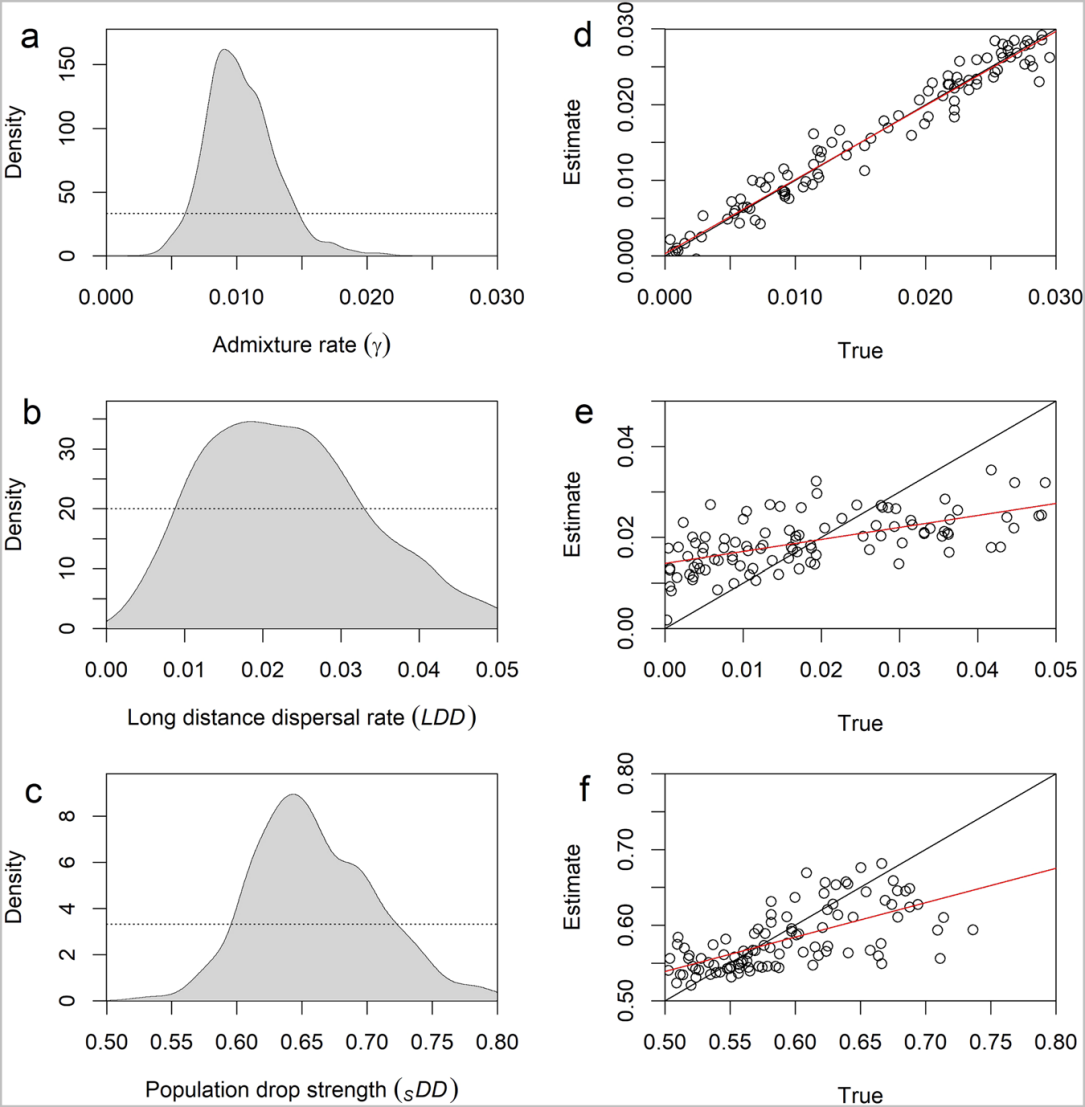
ABC analysis of the most significant parameters. **a–c**) The light gray area indicates the posterior distribution of the parameters, the dotted line shows the prior distribution. **d–f**) Cross-validation results showing the accuracy of the parameter estimation. The dots show the estimated value against the “true” value used in the simulation; the closer the points are to the black line, the more accurate the retrieved true value is. A linear regression between true and estimated values is shown in red.

As displayed in Fig. 2, the most pronounced posterior signals in our analysis are found for the admixture rate (*γ*, estimated to 0.9%, 90% HDI for high density intervals = 0.6–1.5%. PE for prediction error = 0.04), the long-distance dispersal rate (*LDD*, estimated to 2.3%, 90% HDI = 0.6-4.1%, PE = 0.58) and the strength of the population decline (_*S*_*DD*, estimated to 65.4%, 90% HDI = 58.5–74.3%, PE = 0.41). The absolute number of migrants between demes within the farmer layer (*Nm*_*F*_, estimated to 3654; 90% HDI = 1680–5761, PE = 0.45) and within the pastoralist layer (*Nm*_*P*_, estimated to 3,104; 90% HDI = 846–5327, PE = 0.57) also provides interesting information (Table 3), as well as the carrying capacities of farmers and pastoralists (supplementary figures S4.6 and S4.7). The difference in *Nm* values, which is slightly larger for the farmers than for the pastoralist population, is mostly due to the relative difference in population size (*K*_*F*_, estimated to 6448; 90% HDI = 3429–9472, PE = 0.43 and *K*_*P*_, estimated to 5,085; 90% HDI = 1,266-8,165, PE = 0.54) rather than the migration rate (*m*_*F*_, estimated to 0.57; 90% HDI = 0.4–0.77, PE = 0.94 and *m*_*P*_, estimated 0.67; 90% HDI = 0.47–0.84, PE = 0.94). The cross-validation shows that only *γ* can be retrieved with accuracy (Fig. 2), while the precision of the other parameters is more nuanced (Supplementary material 4), as revealed by the large associated HDIs and PE (Table 3). Despite these relatively large confidence intervals, these posterior distributions mostly support our choice for the prior distributions, as the former are always distributed within the latter. The other parameters, the growth rates (*r*_*F*_ *and r*_*P*_), the migration rates (*m*_*F*_ and *m*_*P*_), and the generation when the population decline ends (_*E*_*DD*), do not show clear signals, and we consider them uninformative (*PE* > 0.88). Even though noninformative, these parameters add stochasticity to the complex processes modeled here and we consider them useful to include.

**Table 3:** Parameter estimation results.

	*r*_*F*_	*r*_*P*_	γ	*LDD*	*eDD*	*sDD*	*Nm*_*F*_	*Nm*_*P*_
Prior lower	0.530	0.530	0.000	0.000	230	0.500	400	400
Lower 90% HDI	0.545	0.537	0.006	0.006	233	0.585	1,680	846
Weighted Median	*0.626	*0.608	0.010	0.021	270	*0.654	3433	*3104
Weighted Mean	0.622	0.609	0.010	*0.023	*271	0.66	*3654	3184
Weighted Mode	0.651	0.564	*0.009	0.018	263	0.645	3082	2748
Upper 90% HDI	0.695	0.688	0.015	0.041	311	0.743	5761	5327
Prior upper	0.700	0.700	0.030	0.050	315	0.800	8000	8000
Prediction error Median	0.888	0.810	0.061	0.695	0.986	0.411	0.574	0.578
Prediction error Mean	1.042	0.812	0.059	0.583	0.978	0.530	0.452	0.599
Prediction error Mode	1.401	1.442	0.044	0.880	1.724	0.526	0.682	0.776

Characteristics of the parameter prior distributions for all the scenarios and characteristics of the posterior distribution for the most likely scenario (“*F*&*P*”), with the sampling layer fixed for four samples. The prior distribution for the migration rate (*m*) is 0.4–0.8 and that for the carrying capacity (*K*) is 1000–10,000, leading to a range of 400–8000 for *Nm*. Prediction errors for the median, the mean, and the mode are also given and used to determine which is the best estimator for each parameter independently (*).

## Discussion

Our spatially explicit simulations support the expansion of pastoralists from the Pontic Steppe towards Central Europe during the Bronze Age, with rare—but necessary—long-distance migrations (~2% of the migration events), as well as side by side cohabitation with Central European farmers with limited admixture (1% of the contacts). In addition, the parameter estimation shows that the steppe ancestry components estimated in Bronze Age Central European populations are best explained with local farmer and incoming pastoralist populations having similar sizes; furthermore, the results indicate that both populations suffered an important demographic decline in CE after their initial contact (~65% decline), despite its exact timing and duration remain undetermined.

Previous studies showed that in the context of range expansion, a low level of admixture with a local population is sufficient to explain the high introgression of local genes in the incoming population^1,4,34^. This asymmetrical introgression of neutral genes between a local population at demographic equilibrium and an incoming population in demographic and spatial expansion is due to their different population dynamics, that lead to a rapid dilution of genes from the incoming population into the gene pool of the local population, if admixture is not severely prevented^36^. Consequently, a high steppe ancestry far from the place of origin of pastoralists can be explained by a very low amount of gene flow between both populations, as estimated in our study by an admixture rate of around 1% (i.e., proportion of contacts between individuals of each population resulting in gene flow due to reproduction or community transfer). With higher admixture rates, genes from the initial pastoralist population would have been rapidly diluted in the gene pool of the local populations encountered during their expansion toward CE. This dilution effect is partly counteracted by long-distance dispersal (LDD), which reduces the number of admixture events and thus the amount of local introgression in the dispersing population^38^.

Furthermore, a low level of admixture could potentially explain heterogeneous introgression levels^39^, as observed in CE^12^, western Russia^40^ or in Britain^6^. However, this does not seem to be sufficient here to explain the variance in steppe ancestry, since incorporating an important demographic decline just after the pastoralists reached CE better fit the observed data.

The estimated admixture value means that approximately 1% of contacts between pastoralists and farmers resulted in a change in the lifestyle of one interacting individual or its descendants, thus contributing to mixing genomic legacies from Central European and Pontic steppe populations. The admixture rate is the most informative parameter among those investigated, similar to what has been found when modeling the Neolithic transition with a similar methodology^32^. For comparison, the continuous admixture rate estimated between farmers and pastoralists during the BA is lower than that estimated between hunter-gatherers and farmers during the Neolithic (8.8%^32^). However, contrary to our best scenario of cohabitation during the BA, admixture between hunter-gatherers and early Neolithic farmers was simulated during a much shorter cohabitation period before the disappearance of the foraging lifestyle due to competition with the farmers. The effective cohabitation time in the current study corresponds to a minimum of around 60 generations while it was around 15 generations in the simulation of the Neolithic^32^. Note that those values vary across simulations depending on the combination of parameters. This explains why a lower admixture rate over a longer period (this study) leads to similar introgression as that observed with a higher admixture rate over a shorter period (simulations of the Neolithic transition^32^).

Our results support the occurrence of LDD at a low rate (2.3%), meaning that approximately 2% of migrations would have occurred over long distances. LDD helped pastoralists reach the sampled locations in CE at the corresponding dates (Supplementary material 5). These LDD events would have facilitated the rapid spread of pastoralists from the Pontic steppe to CE and would have prevented the steppe ancestry from being rapidly diluted in the local gene pool. A high admixture rate may compensate for the absence of LDD, as it would permit the faster spread of pastoralists due to the incorporation of many local farmers along the dispersal route but it would also results in lower steppe ancestry proportions, which is incompatible with observed data. Our LDD estimate is about half of the estimate attributed to European hunter gatherers during the Last Glacial Maximum (LGM) when LDD only occurs on already inhabited lands (4.4%)^41^.

We estimated a large number of migrants between demes (*Nm*) for both the farmers and pastoralists (*Nm*_*F*_ ≈1827 and *Nm*_*P*_ ≈1552 diploid individuals), indicating that gene flow is extensive within both populations. These values tend to be in the higher range compared to other *Nm* estimations for human populations from genetic data^32,41–44^. However, we will not interpret these absolute values extensively, since our cross-validation shows that the estimates for the parameter *Nm* lack precision and that there are many differences across studies, such as deme sizes, as well as spatial and temporal contexts. What is interesting, however, is the impossibility to differentiate between the number of migrants estimated from the farmer and pastoralist populations. This suggests that the “massive” change that occurred in the genomes of CE populations during the BA does not necessarily mean that local farmers were overwhelmed by a large immigrant population and that a continuous process of migration without much admixture could also explain this genomic pattern.

A straightforward interpretation of a scenario of cohabitation with limited but ongoing admixture between the CE farmers and immigrant pastoralists from the Pontic steppe would mean that they lived side by side after their contact while exchanging a limited number of genes. It should be noted that limited gene flow between populations does not mean limited cultural contacts and exchanges but the purpose of our modeling approach is to simulate biological processes, not cultural processes. It is indeed criticizable to associate material cultures to biological entities (i.e., genetically differentiable populations) because they do not necessarily overlap^20,45–48^.

The model without direct competition, which is supported by our analysis, does not contradict the different economic subsistence strategies of pastoralists and farmers. Populations with different lifestyles could indeed cooperate, leading to their coexistence over long periods, which would not appear as a cultural “change” from the archeological point of view but would be associated with a cultural complex (diverse combinations of common materials from geographically distant populations), such as the Corded Ware complex (CWC) associated with the populations analysed in our study. However, we have tested only two extreme situations (with and without full competition) and cannot exclude more nuanced processes of competition between local farmers and incoming pastoralists in CE.

Our results thus do not contradict the proposal that moving people can result in a homogenization of material culture, such as the CWC, while communities remain distinct for specific combinations of cultural characteristics/material^20^. The fact that a strong, fast, and well-delimited cultural change associated with cultures from the steppes did not appear in CE at the same time as large genomic components related to a steppe ancestry could be explained by the coexistence of pastoralists and farmers on a large geographical scale.

Our model is a simplified representation of societal and biological mechanisms related to processes that would have occurred in CE during the BA. It is meant to improve the understanding of those processes as a complement to other approaches. Despite improving on the realism of panmictic models by incorporating spatially explicit features, our modeling framework still has limitations that are worth mentioning. First, we tested only a limited number of scenarios. For instance, we did not consider the spatiotemporal heterogeneity of the environment and other processes associated with population dynamics, such as the temporal variance of the population growth rate, the effect of matrilocality or patrilocality (see^5^), and the effect of social structure^49^ (e.g., the presence of elites). Second, we used ancestry proportions as proxies of genomic data to estimate demographic parameters. The use of ancestral components somewhat limits the molecular information available from paleogenomes for making demographic inferences, but the dataset used fits the kind of information that is simulated by the program SPLATCHE3 particularly well (i.e., the genomic proportions in the samples from different dates and locations coming from two spatially different source populations). There is a strong correlation between those two quantities (i.e., estimated ancestry components and simulated genomic proportions, Supplementary material 6). Third, although the dataset analysed is constituted of 34 paleogenomes, it is still a limited sample of the area and period of interest, and the question of whether it is sufficient to obtain a representative estimate of the various genomic components arises. Despite a larger number of paleogenomes from the area is available from various publications (e.g.,^6,13,14,50,51^), we were limited by the number of population samples that we can used in our study namely due to two technical constraints: the impossibility of obtaining ancestry proportions in the same deme at several different times with SPLATCHE3 and the exponential increase in combinations of sampling layers (2^*n*^ combinations where *n* is the number of population samples, multiplied by the number of simulations). Nevertheless, following studies^6,13^ confirmed the general pattern described in Haak et al.^12^ about a sudden appearance of large steppe ancestry components in CE during the BA and we verified the representativeness of the data analysed against a larger dataset available from multiple sources (Supplementary material 7), thus consolidating the relevance of our work. The use of the ancestry component as we do here is a promising solution to cope with the issues related to data aggregation which results from the heterogeneity in sequencing coverage (e.g.,^32^) and it opens new avenues in the spatial analysis of ancient DNA.

While simulations of expansion from the Pontic steppes have been modeled based on allele frequencies^33,34^, as far as we know our study is the first attempt to model complex population movements and turnovers during the BA in Western Eurasia by incorporating paleogenomic data. Further model extensions can be devised, such as implementing spatiotemporal variance of the admixture rate between the local and incoming populations, by extending the spatiotemporal scale of the paleogenomic dataset, from the Pontic steppe to the east and the British Isles to the west.

Our results show that a large steppe ancestry component in Central European BA populations can be explained by a model of pastoralist expansion with long-distance dispersal combined to a limited gene flow with local populations. This low admixture rate is necessary to avoid a fast dilution of steppe ancestry in the gene pool of local populations encountered by the Pastoralists during their migration toward Central Europe. Furthermore, we found that a further demographic decline is necessary to explain the spatiotemporal heterogeneity of this steppe ancestry among the samples analysed. Note that the observed heterogeneity in steppe ancestry could be partly dependent on the samples, and additional data are needed to further confirm this observation. We estimated the amplitude of this demographic decline to be very strong (between 60 and 75%), but we did not obtain much information about its start and duration (the parameter *eDD* was uninformative). We started this work with the hypothesis that *Y. pestis* may have been the cause of a population decline, but the literature concerning the epoch approximately 4500 BP reveals that this period is marked by climatic, social, migratory, and economic changes that could potentially have affected the demography and genomic background of BA populations. Below, we discuss a list of possible causes that could explain a large population decline occurring at approximately 4500–4,000 BP in CE, encompassing the time when the samples in the dataset we used are dated from.

- *Disease:* Our initial hypothesis was that early BA populations from the steppes would have been resistant to an ancient strain of *Y. pestis*, while Central European populations would have been vulnerable. We thought that this could have contributed to the sudden appearance of large inputs from populations from the steppes to the genomic profiles of Central European populations. Under the hypothesis that the spread of pastoralist populations was favored by a disease that decimated local farmers only, we were expecting a demographic decline in the farming population but not in the pastoralist population, corresponding to scenario “*F,*” which received low support (16.7% posterior probability). If the demographic decline observed in the scenario with the highest support (“*F*&*P*”, 54.5%) had been due to a disease, it would thus have affected both populations, not only the farmers. Note that scenario “*P,*” in which only the pastoralist population is affected by a demographic decline, is as probable as scenario “*F*,” with a posterior probability of 16.4%.
- *Climate:* Brutal climatic events, such as the one occurring approximately 4200 BP may have had major impacts on human populations, including in the European continent^52–57^ and its effects could have started hundreds of years earlier. A demographic decline starting around 4450 BP, as in our simulations, could potentially be explained by climatic events degrading environmental conditions and leading to reduced resources accessible to both the pastoralists and farmers.
- *Resource depletion:* The exploitation of the ecosystem, together with growing material needs, could lead to the fast degradation of the environment and to a demographic collapse. Technological improvements can provide an enhanced carrying capacity in the short term, although eroding the resources and reducing the carrying capacity in the long term^58^. Ecosystem loss might overcome its resilience due to increasing stress induced by humans, leading to a critical disequilibrium that may cause the collapse of the whole system^59^. Economic stratification can also independently lead to a population decline by inducing economic inequalities and the loss of resources^60,61^. For instance, Svizzero^62^ showed that the BA prosperity in CE, due to metalworking, required a large amount of primary resources that led to an economic crisis called the “Dutch disease.” This may explain the later collapse of the Únětice Culture, which is more recent than the dataset used in this study. In our case study, the populations could have affected their common resources in the long term, affecting both carrying capacities (scenario “*F*&*P*”). This could be considered a form of indirect competition equally affecting both parties.
- *Direct conflicts:* Direct conflicts between farmers and pastoralists could explain the demographic decrease in CE during the BA. These events could affect both populations or only one population (in the case of a larger carrying capacity and/or technical advantage for the other population). It was suggested that populations associated with the Bell Beaker culture, also carriers of a large proportion of steppe ancestries, are responsible for population replacement at approximately 4500 BP on the British Islands^6^. Because Bell Beaker communities were also present in CE around this period, they may have been the cause of the demographic decrease in this area. However, this is speculative, as we have no evidence supporting such conflicting events in CE.
- *Biased sex ratio of reproducers:* Another social hypothesis involves a biased sex ratio among reproducers. Indeed, a biased sex ratio among individuals contributing to reproduction, passing from 1.0 to 0.25, would result in an effective population size reduction of 60%^63,64^, while the census population size would remain the same. Goldberg et al.^65^ suggested BA migration driven by males (5-14 males for one female) approximately 5000 BP. Moreover, Heyer et al.^66^ showed that the transmission of reproductive success can greatly reduce the effective population size in one or the other sex, an effect that could be detected by analysing patterns of genetic variation on the Y and X chromosomes. While we analysed autosomal genetic patterns, the inference of a large population decrease in CE may be explained by biased sex ratio or by changes in cultural practice affecting each sex differently^67,68^.

Our spatially explicit modeling of population interactions in Central Europe during the Bronze Age allows us to reconcile paleogenomics and archeological observations. Indeed, our results show that the rapid genomic changes that occurred in Central European populations during this period do not necessarily indicate one large population migration from the Pontic steppe, for which archeological evidence is missing^3^. Continuous gene flow, including both short- and rare long-distance movements from Eastern populations, leading to settlement alongside local communities with limited genetic admixture, is the most likely scenario among those investigated. This scenario does not contradict locally heterogeneous material cultures within a common cultural complex at a larger scale, such as the Corded Ware. Moreover, our results do not support our former assumption that the spread of pastoralists could have been favored by the joint diffusion of pathogens to which indigenous populations would have been less resistant. Additional research taking into account paleogenomic data from the whole European continent would help in further understanding this key period that saw major population and cultural changes^6,8,11,27,69^.

## Methods

### Spatially explicit simulation framework

Spatially explicit simulations of the interactions between sedentary Central European populations, hereafter referred to as farmers (“*F*”), and immigrant populations from the Pontic steppe, hereafter referred to as pastoralists (“*P*”), were performed with a version of the program SPLATCHE3^35^ allowing for LDD with the two-layer option. This program simulates the demography and migration of populations through space and time by taking into account the type of interactions between them (e.g., competition and admixture), various types of migration (short- and long-distance), the spatiotemporal heterogeneity of the environment, and the resulting molecular diversity based on a serial coalescent approach.

**Fig. 3:**
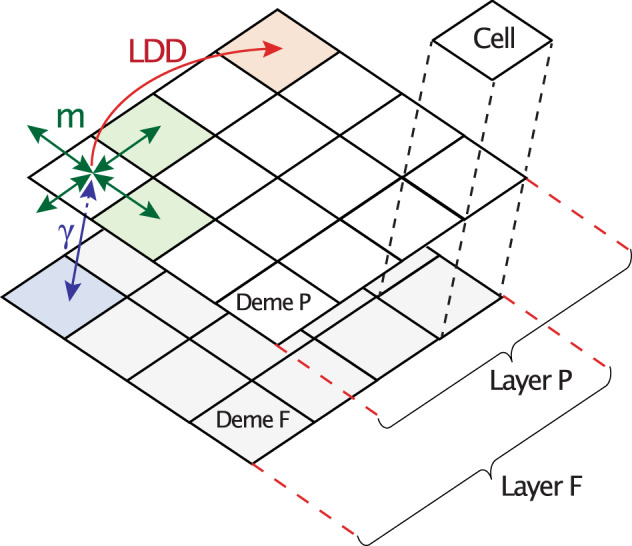
Schematic representation of the modeling framework. Explanation about the computation of the various parameters presented in this figure are given in the Methods section.

SPLATCHE3 represents each population (pastoralists or farmers) as a layer of interconnected demes of 100 by 100 *km* distributed over a realistic map of Europe (Fig. 1). The map represents approximately 6,000 *km* along the longitudinal axis and 5000 *km* along the latitudinal axis. The map is projected in a PlateCarree WGS84 format. The simulation starts with a “demographic step,” consisting of the expansion of farmers from the Near East at the onset of the Neolithic transition, taking 10,000 BP (e.g.,^70,71^) in the first grid of demes (layer *F*). This is followed by the expansion of pastoralists from the Northern Caucasus area starting at the end of the Neolithic (5600 BP)^10^ in a second superimposed grid of demes (layer *P)*. We started the expansion of our first simulated pastoralists at 5600 BP. In this manner, by 5300 BP the extent of these simulated pastoralist populations is similar to the actual Yamnaya cultural horizon. Generation 0—the beginning of a simulation—corresponds to the onset of the Neolithic, while the present corresponds to generation 400—the end of a simulation—assuming a generation time of 25 years. The model takes into account direct competition and admixture between the two layers and the effect of a demographic decline in every demes of each layer after their initial contact in CE. As shown schematically in Fig. 3, within each layer, gene flow is simulated among neighboring demes by using the migration rate (parameter *m*) and the long-distance dispersal rate (parameter *LDD*). Short-distance migration cover 100 *km* while long-distance migration covers on average 800 *km*. The population density within each deme increases at the growth rate (parameter *r*) until reaching a carrying capacity (parameter *K*). The population sizes and carrying capacities in SPLATCHE3 are given by the number of gene copies. Each population (pastoralists or farmers) has its own demographic and migratory characteristics. The “assimilation” model of SPLATCHE3 was used^35^ where the admixture rate regulates the gene flow between demes from the same location and different layers (parameter γ), going from 0 (no mixing) to 1 (full mixing). Moreover, SPLATCHE3 takes into account intrademic competition, which is represented by the effect of carrying capacity on the logistic growth regulation within each deme of both layers. In addition, the Lotka–Voltera competition model^72,73^ was used to test for direct competition between pastoralists and farmers, which may be due to warfare or common resource exploitation. The parameter “CompetitionModel” was set to 0 without competition or 1 with competition.

For the various scenarios described below, 30,000 demographic simulations were performed, where each simulation used a different combination of parameters taken independently from prior distributions (Table 3). The prior ranges used for the farmer layer are based on ranges from Silva et al.^32^. In the absence of further information, parameters of the pastoralist layer were taken from the same prior distributions as those of the farmers (Table 4). After the demographic step, the “genetic step” starts, which consists of the simulation of 50 independent autosomal SNPs from different populations in CE using the serial coalescent algorithm. The sizes, locations and ages of the genetic samples are similar to those of the observed dataset published by Haak et al.^12^ (see Table 1). For each demographic simulation, one stochastic genetic simulation is performed for each simulated SNP based on probabilistic numbers computed during the demographic step (see^35^ for details about the algorithm).

**Table 4:** SPLATCHE3 parameters.

Parameter	Description
Demes size	100 *km* * 100 *km*
Starting layer 1	10 Ky BP (generation 0/400) in the Fertile Crescent
Starting layer 2	5.6 Ky BP (generation 176/400) in Northern Caucasus
Model used	100 (CompetitionModel = 0 without competition or 1 with competition)
LDD dispersal type	Long-distance dispersal toward all cells (whether empty or occupied)
LDD gamma shape	1.209
LDD gamma scale	0.15046
Proportion of LDD events	[0.0–0.05] applied to both layers
Admixture rate	[0.0–0.03] identical in both directions
Carrying capacity	[1000–10000] applied to both layers independently
Migration rate	[0.4–0.8] applied to both layers independently
Growth rate	[0.53–0.7] applied to both layers independently
SNP per paleogenome	50

List of the input parameter values used in SPLATCHE3 for all the simulations. The four scenarios differ in the value of the demographic decline duration (eDD) and intensity (sDD).

### Description of the scenarios investigated

We explored the change in the genomic makeup of Central European populations during the BA. We primarily tested whether this change could have been facilitated by a pandemic brought by the incoming population that affected the local population only. We hypothesized that a strain of *Y. pestis* could possibly be the responsible pathogenic agent.

Accordingly, we designed four different scenarios representing different alternative situations: i) the “*control*” scenario, in which the two populations mix without any subsequent population decline, ii) the “*F*” scenario, in which the two populations mix, with a further population decline in the demes of the farmer layer only (which could result from a disease carried by the pastoralist population to which farmers were less resistant), iii) the “*P*” scenario, in which the two populations mix, with a further population decline in the demes of the pastoralist layer only (which could result from a disease present in the farmer population to which the pastoralists were less resistant) and iv) the “*F*&*P*” scenario, in which the two populations mix, with further population declines in the demes of both the farmer and the pastoralist layers (which could result from a disease affecting both populations in a similar way). Note that this population decline could be due to any other factor responsible for reducing population size, not only pathogens.

### Demographic decline parameters

In the scenarios in which population decline occurs after contact between farmers and pastoralists in CE, two parameters are involved: the generation at which the population decline ends (in generations, _*E*_*DD*) and its strength as a ratio of *K* (_*S*_*DD*).

The starting date of the population decline is fixed at generation 222 (~ 4450 BP), corresponding to the period when a pandemic caused by a strain of *Y. pestis* may have spread in Europe along with the migration of steppe populations^30,74^. Because Valtuena et al.^30^ hypothesized that the plague could have spread from the area around Estonia, we chose to fix the starting date of the decline to an average value between the three sample sites where the bacillus was found in this area^74^.

The chosen prior for the reduction in the population size due to plague is a subset (50–80%) of the lethal range proposed by the World Health Organization (WHO) without medical treatments (30–100%^75^). The plague is less lethal in its bubonic form (30–60% death rate) than in its pulmonary form (near 100% lethal rate). A subset of values was used because on the one hand, lower values would render the various scenarios difficult to differentiate, while on the other hand, higher values would produce simulations with empty demes at corresponding sampling times, a situation we know does not fit current data. Moreover, the upper limit of the prior is in accordance with the maximum historical values estimated for the Black Death (80% reduction of the population size)^76^.

### Long-distance dispersal (LDD) parameters

In SPLATCHE3, the direction of the LDD is randomly drawn first, and then the distance traveled by the migrant is drawn from a *gamma* distribution defined by a shape parameter, α (also called *k*), and a scale parameter, *θ* (corresponding to the inverse of the rate parameter *β* = 1/*θ*). The maximum prior probability for an emigrant to travel more than one deme away from its current deme (LDD event), as well as the average number of demes traveled by migrants (*µ*) and the *gamma* distribution shape parameter (*α*) values were retrieved from Alves et al.^41^. The other variables were corrected to take into account the difference in deme size (Supplementary material 8), because in Alves et al.^41^, LDD parameters are given for demes of 150 *km* by 150 *km*, whereas here, we used demes of 100 *km* by 100* km*. On average, LDD events cover 800 *km*. We used a version of SPLATCHE3 allowing for LDD in both population layer by using the demographic model 100.

### Simulation optimization

We proceeded to a series of simulations to optimize the exploration of the parameter space and to minimize the number of parameter combinations that would result in an output incompatible with the observed data. This step was necessary due to the long duration of the simulations and the numerous scenarios to be tested. The exploratory simulations showed that an admixture rate larger than 0.03 is never compatible with observations, so we decided to reduce the prior range accordingly (0.0–0.03 instead of 0.0–1.0). Moreover, the exploratory simulations qualitatively showed that pastoralist populations have very little probability of reaching CE without long-distance dispersal (LDD), except with the help of specific combinations of demographic parameters, such as a large admixture rate. We therefore decided to use a prior distribution for the LDD rate (0–5%), which includes the absence of LDD, as we do for the admixture rate (0–3%).

### Location and timing of samples

For each simulation, we sampled 10 populations (described in Table 1) as follows. Dates given in BP were converted to calBC with the help of the OxCal program version 4.3^77^ by using the IntCal13 atmospheric curve^78^ (Supplementary material 9). Conversion from calBC to calBP required the addition of 1950 years. The dates were then transformed into *t* generations of 25 years after 10,000 BP (generation 0). For each simulation and each sampling location, sampling dates were randomly drawn from a uniform prior distribution, with the limits taken as the average minimum and maximum dates of all the paleogenomes from the same location.

The sampled locations of the simulated paleogenomes in the virtual map are approximated from their real coordinates (Fig. 1). Some of the sampled locations do not exactly match their original locations for a technical reason: SPLATCHE3 only allows to compute genomic proportions from one deme at one generation time. Consequently, we selected the closest deme to reduce the error in the distance from the observed data. Thus, the samples are always picked in the deme corresponding to the observed data or directly adjacent to it.

### Sample layer optimization

SPLATCHE3 uses virtual layers to represent the two populations, which are differentiated by their lifestyle (pastoralists and farmers). Because the Central European BA is a culturally complex period, the attribution of observed samples to one or the other lifestyle is not trivial, because it depends on the related archeological context, which can be ambiguous^79^. This choice matters because sampling in different population layers (farmer or pastoralist) may result in different steppe ancestry proportions. Obviously, samples coming from the farmer layer tend to show lower steppe ancestry proportions than samples taken from the pastoralist layer and not considering this ambiguity could have affected the estimation of the admixture rate. To account for this issue and avoid making any a priori decisions on the lifestyles associated with the observed samples, we collected samples from both layers, and then we made all 1024 combinations of the 10 samples by alternatively taking each sample from one of the two layers (2^10^).

To reduce the number of improbable simulations and save computational time, we used the layer id (either farmer or pastoralist) as an input parameter for the ABC analysis to estimate the most likely layer for each sample, and we fixed this layer for the samples in which the signal was sufficiently unambiguous. A value of 0 indicates sampling in the farmer layer, while 1 indicates sampling in the pastoralist layer. When the posterior distribution of a sample was above 0.5 for the lower value of the 99% HDI, we took that sample in the pastoralist layer, while we took it in the farmer layer when the upper value of 99% HDI was under 0.5. When the four demographic scenarios without competition allow us to fix a given sample to the same layer, we fixed this layer for that sample. By using this method, we were able to define the most likely layer for four (out of 10) observed samples (Supplementary material 3): “Esperstedt_MN” and “Hungary_Gamba_CA” were assigned to the farmers, a result in accordance with archeological knowledge which associates them, respectively, to the Middle Neolithic and the Chalcolithic, while “Corded_Ware_LN” and “Karsdorf_LN” were assigned to the pastoralist layer, despite having been archeologically associated with the Late Neolithic; however, the distinction between Late Neolithic and BA archeological contexts may be ambiguous. Note that “Esperstedt_MN” was never able to be sampled in the pastoralist layer due to the demographic filter (see below).

### Demographic filter

Each set of demographic parameters leads to many sampling combinations, whether the samples may be drawn from both population layers or from a fixed one (as for the four abovementioned examples). By performing 30,000 simulations (each one made of different sets of demographic parameters) for each scenario, we ultimately have a maximum possible number of 30,000 × 2^10^ = 30,720,000 simulation results without fixing the sampling layers or 30,000 × 2^6^ = 1,920,000 for the analyses in which the four samples were fixed. However, those maxima are never met due to specific sets of demographic parameters being incompatible with all the sampling dates and locations. Indeed, all the demes where sampling occurs need to be populated at the corresponding sampling time. Thus, those simulations that do not allow drawing at least one sample (out of ten samples) from a deme are not considered for the next step (the ABC estimation). This serves as a demographic filter, and has the advantage of discarding the less probable combinations of parameters that are not in accordance with archeological records (i.e., where simulated demes are still empty despite observed data existing at the corresponding time). Moreover, some sets of demographic parameters lead to the possibility of sampling in one layer but not in the other, leading to a drastic decrease in the final number of layer combinations.

The number of remaining simulations after the demographic filtering can be counted in millions for each scenario without competition [1,000,000–8,000,000] and in hundreds of thousands for each scenario with competition [100,000–700,000]. Finally, a subset of 100,000 simulations is randomly drawn from the whole set of simulation results for each scenario for further ABC estimation, as datasets of millions of simulations are too large for the “abc” R package to run in an acceptable time. We checked that the results are robust to the random choice of 100,000 simulations (Supplementary material 10).

### Steppe-ancestry proportions

We used published data of 10 population samples composed of 34 paleogenomes^12^ dating from the Bronze Age in Central Europe, published by Haak et al.^12^, to evaluate the probability of our scenarios and to estimate the parameters. This dataset has the advantage of providing unbiased admixture proportions from steppe pastoralists and farmers that represent similar quantities, although computed differently, to the genetic proportions outputted by SPLATCHE3 (Supplementary material 6). From the original dataset, we kept only the samples dating after the onset of the Yamnaya Cultural Complex (YCC) in the Pontic steppe, as earlier samples are uninformative about the relation between Central European populations and the YCC. Looking at the timeline of those 10 populations, those associated with the Corded Ware cultural complex (CWC) are the first to show a large amount of steppe ancestry in CE. The estimated proportion of steppe ancestry tends to decrease in populations associated with later cultures (Table 1).

The Haak et al.^12^ study estimated three genomic ancestry components in the paleogenomic dataset: the first one associated with pre-Neolithic hunter gatherers, the second one associated with Neolithic farmers, and the third one associated with Yamnaya pastoralists from the Pontic steppe. Here, we focus on the genomic ancestry component from the pastoralists and consider the two others to be jointly related to local farmers, mixing both ancestries from previous hunter-gatherers and later incoming Neolithic farmers.

For each scenario, 50 independent autosomal loci were simulated for 30,000 simulations consisting of different combinations of demographic parameters taken from their respective priors. For each locus, we computed the proportion of alleles arriving in CE with the pastoralist layer from its source in the Pontic steppe using the “.prop” output of SPLATCHE3, similar to what was done previously for the Neolithic transition to compute the genomic contribution of farmers and hunter-gatherers^32^. The pastoralist proportion computed in each of the 10 “population samples” was averaged over the 50 loci and served as 10 statistics for the ABC estimation. Note that some of the “population samples” are represented by a single genome (Table 1). We verified that 50 independent loci were enough to obtain accurate estimates of the pastoralist proportions in the samples with SPLATCHE3 (Supplementary material 11).

### ABC analyses for model and parameter estimation

All the approximate Bayesian computation (ABC) analyses were performed using the package “abc” (version 2.1) in R^80^. Except when specified otherwise, the same parameters were used for all the analyses: the “neuralnet” method was applied with a tolerance equal to 0.01 and 100 replicates for the cross-validation procedures. A tolerance rate of 0.01 was the best compromise between the accuracy and robustness of the results (Supplementary material 12), while more than 100 replicates required considerable computation time for little improvement in the cross-validation analyses. We used the posterior probabilities from the confusion matrix (Supplementary material 2) to calculate the probability of recovery (PR) of the most likely scenario, i.e., the proportion of times the most likely scenario is identified while being generated by itself over the proportion of times it is identified regardless of the scenario that generated it. We also provide pairwise Bayes factor (BF_12_) for model *M*_*1*_ against model *M*_*2*_ and interpret its value using the scale by^81^ adjusted from^82^: with BF_12_ = 1-3, the evidence in favor of *M*_*1*_ is “anecdotal”, with BF_12_ = 3–10 it is “moderate”, “strong” with BF_12_ = 10–30, “very strong” with BF_12_ = 30–100 and “extreme” with BF_12_ > 100. To evaluate the precision of the parameter estimation, we used the prediction error (PE) from the “abc” package, computed as: ${PE}=\frac{{\sum }_{i}{\left(\,{\,\widetilde{\theta }}_{i}-{\theta }_{i}\right)}^{2}}{{Var}({\theta }_{i})}$ where ${\theta }_{i}$ is the true parameter value of the i^th^ replicate and ${\widetilde{\theta }}_{i}$ is the estimated parameter value estimated by either the mode, the median or the mean of the posterior distribution.

### Reporting Summary

Further information on research design is available in the Nature Research Reporting Summary linked to this article.

## Supplementary information

Supplementary material

Reporting Summary

## Data Availability

The data analysed were originally published in^12^ and are listed in Table 1.

## Code availability

We provide the list of parameter values for SPLATCHE3 in Table 4. The input datasets as well as the executable are publicly available in Zenodo with DOI number 10.5281/zenodo.5205827.

## References

[1] Currat M, Excoffier L (2005). The effect of the Neolithic expansion on European molecular diversity. Proc. R. Soc. B Biol. Sci..

[2] Skoglund P (2012). Origins and genetic legacy of Neolithic farmers and hunter-gatherers in Europe. Science.

[3] Ammerman, A. J. & Cavalli-Sforza, L. L. *The Neolithic Transition and the Genetics of Populations in Europe**:* (Princeton University Press, 1984). 10.1515/9781400853113.

[4] Chikhi L, Nichols RA, Barbujani G, Beaumont MA (2002). Y genetic data support the Neolithic demic diffusion model. Proc. Natl Acad. Sci..

[5] Rasteiro R, Bouttier P-A, Sousa VC, Chikhi L (2012). Investigating sex-biased migration during the Neolithic transition in Europe, using an explicit spatial simulation framework. Proc. R. Soc. B Biol. Sci..

[6] Olalde I (2018). The Beaker phenomenon and the genomic transformation of northwest Europe. Nature.

[7] Martiniano R (2017). The population genomics of archaeological transition in west Iberia: Investigation of ancient substructure using imputation and haplotype-based methods. PLOS Genet.

[8] Olalde I (2019). The genomic history of the Iberian Peninsula over the past 8000 years. Science.

[9] Gimbutas, M. The Indo-Europeanization of Europe: the intrusion of steppe pastoralists from south Russia and the transformation of Old Europe. *Word.* **44**, 205–222 (1993).

[10] Morgunova, N. Chronology and periodization of the pit-grave culture in the area between the Volga and Ural rivers based on 14c dating and paleopedological research. *Radiocarbon* **55**, 1286–1296 (2013).

[11] Preda-Bǎlǎnicǎ B, Frînculeasa A, Heyd V (2020). The Yamnaya impact North of the lower Danube: a tale of newcomers and locals. Bull. Soci.été Pr.éhistorique Fr..

[12] Haak W (2015). Massive migration from the steppe was a source for Indo-European languages in Europe. Nature.

[13] Allentoft ME (2015). Population genomics of Bronze Age Eurasia. Nature.

[14] Narasimhan VM (2019). The formation of human populations in South and Central Asia. Science.

[15] Kristiansen K (1989). Prehistoric migrations—the case of the single grave and corded ware cultures. J. Dan. Archaeol..

[16] Heyd V (2017). Kossinna’s smile. Antiquity.

[17] Kristiansen K (2017). Re-theorising mobility and the formation of culture and language among the Corded Ware Culture in Europe. Antiquity.

[18] Klejn LS (2017). The steppe hypothesis of indo-european origins remains to be proven. Acta Archaeol..

[19] Furholt, M. Re-integrating archaeology: a contribution to aDNA studies and the migration discourse on the 3rd millennium BC in Europe. *Proc. Prehist. Soc*. **85**, 1–15 (2019).

[20] Furholt M (2018). Massive Migrations? The Impact of Recent aDNA Studies on our View of Third Millennium. Eur. Eur. J. Archaeol..

[21] Juras A (2018). Mitochondrial genomes reveal an east to west cline of steppe ancestry in Corded Ware populations. Sci. Rep..

[22] Horvath CB (2015). R1A subclades and Bronze Age migrations on the Eurasian steppes. Eur. Sci. J..

[23] Marciniak S, Perry GH (2017). Harnessing ancient genomes to study the history of human adaptation. Nat. Rev. Genet..

[24] Mathieson I (2018). The genomic history of southeastern Europe. Nature.

[25] Bocquet-Appel J-P (2011). The agricultural demographic transition during and after the agriculture inventions. Curr. Anthropol..

[26] Porčić M, Blagojević T, Stefanović S (2016). Demography of the early Neolithic population in Central Balkans: population dynamics reconstruction using summed radiocarbon probability distributions. PLoS ONE.

[27] Shennan S (2013). Regional population collapse followed initial agriculture booms in mid-Holocene Europe. Nat. Commun..

[28] Rasmussen S (2015). Early Divergent Strains of Yersinia pestis in Eurasia 5,000 Years Ago. Cell.

[29] Demeure CE (2019). Yersinia pestis and plague: an updated view on evolution, virulence determinants, immune subversion, vaccination, and diagnostics. Genes Immun..

[30] Andrades Valtueña A (2017). The stone age plague and its persistence in Eurasia. Curr. Biol..

[31] Spyrou MA (2018). Analysis of 3800-year-old Yersinia pestis genomes suggests Bronze Age origin for bubonic plague. Nat. Commun..

[32] Silva NM, Rio J, Kreutzer S, Papageorgopoulou C, Currat M (2018). Bayesian estimation of partial population continuity using ancient DNA and spatially explicit simulations. Evol. Appl..

[33] Rendine S, Piazza A, Cavalli-Sforza LL (1986). Simulation and separation by principal components of multiple demic expansions in Europe. Am. Nat..

[34] Barbujani G, Sokal RR, Oden NL (1995). Indo-European origins: a computer-simulation test of five hypotheses. *Am*. J. Phys. Anthropol..

[35] Currat, M., Arenas, M., Quilodràn, C. S., Excoffier, L. & Ray, N. SPLATCHE3: simulation of serial genetic data under spatially explicit evolutionary scenarios including long-distance dispersal. *Bioinformatics*. **35**, 4480–4483 (2019). 10.1093/bioinformatics/btz311 PMC6821363 31077292

[36] Currat, M., Ruedi, M., Petit, R. J. & Excoffier, L. The hidden side of invasions: massive introgression by local genes. *Evolution.* **62**, 1908–1920 (2008). 10.1111/j.1558-5646.2008.00413.x 18452573

[37] Beaumont MA, Zhang W, Balding DJ (2002). Approximate Bayesian computation in population genetics. Genetics.

[38] Amorim CEG (2017). Long-distance dispersal suppresses introgression of local alleles during range expansions. Heredity.

[39] Quilodrán CS, Tsoupas A, Currat M (2020). The spatial signature of introgression after a biological invasion with hybridization. Front. Ecol. Evol..

[40] Saag L (2021). Genetic ancestry changes in Stone to Bronze Age transition in the East European plain. Sci. Adv..

[41] Alves I (2016). Long-distance dispersal shaped patterns of human genetic diversity in Eurasia. Mol. Biol. Evol..

[42] Pimenta J, Lopes AM, Comas D, Amorim A, Arenas M (2017). Evaluating the Neolithic expansion at both shores of the Mediterranean Sea. Mol. Biol. Evol..

[43] Currat M, Poloni ES, Sanchez-Mazas A (2010). Human genetic differentiation across the Strait of Gibraltar. BMC Evol. Biol..

[44] Di, D., Sanchez-Mazas, A. & Currat, M. Computer simulation of human leukocyte antigen genes supports two main routes of colonization by human populations in East Asia. *BMC Evol. Biol*. **15**, 1908–1920 (2015). 10.1186/s12862-015-0512-0 PMC4632674 26530905

[45] Brandt G (2013). Ancient DNA reveals key stages in the formation of Central European mitochondrial genetic diversity. Science.

[46] Cavalli-Sforza LL, Menozzi P, Piazza A (1993). Demic expansions and human evolution. Sci. N. Ser..

[47] Veeramah KR (2018). The importance of fine-scale studies for integrating paleogenomics and archaeology. Curr. Opin. Genet. Dev..

[48] Zvelebil M (2001). The agricultural transition and the origins of Neolithic society in. Eur. Doc. Praehist..

[49] Parreira BR, Chikhi L (2015). On some genetic consequences of social structure, mating systems, dispersal, and sampling. Proc. Natl Acad. Sci..

[50] Mathieson I (2015). Genome-wide patterns of selection in 230 ancient Eurasians. Nature.

[51] Lipson M (2017). Parallel palaeogenomic transects reveal complex genetic history of early European farmers. Nature.

[52] deMenocal PB (2001). Cultural Responses to Climate Change During the Late Holocene. Science.

[53] Magny M (2004). Holocene climate variability as reflected by mid-European lake-level fluctuations and its probable impact on prehistoric human settlements. Quat. Int..

[54] Smith, H. B. *Re-examining Late Chalcolithic Cultural Collapse in South-East Europe* (Arkansas, 2013).

[55] Wang J (2016). The abrupt climate change near 4,400 yr BP on the cultural transition in Yuchisi, China and its global linkage. Sci. Rep..

[56] Watanabe, T. K., Watanabe, T., Yamazaki, A. & Pfeiffer, M. Oman corals suggest that a stronger winter shamal season caused the Akkadian Empire (Mesopotamia) collapse. *Geology.* **47**, *1*141–1145 (2019).

[57] Wiener MH (2014). The interaction of climate change and agency in the collapse of civilizations ca. 2300–2000 BC. Radiocarbon.

[58] Rees WE (1996). Revisiting carrying capacity: area-based indicators of sustainability. Popul. Environ..

[59] Arrow K (1995). Economic growth, carrying capacity, and the environment. Science.

[60] Motesharrei S, Rivas J, Kalnay E (2014). Human and nature dynamics (HANDY): Modeling inequality and use of resources in the collapse or sustainability of societies. Ecol. Econ..

[61] Svizzero, S. & Tisdell, C. A. The Demise of the Únĕtice Culture due to the Reduced Availability of Natural Resources for Bronze Production. *Int. J. Res. Sociol. Anthropol*. **4**, 33 (2018).

[62] Svizzero S (2015). The collapse of the Únětice culture: economic explanation based on the “Dutch disease”. *Czech*. J. Soc. Sci. Bus. Econ..

[63] Wright S (1933). Inbreeding and Homozygosis. Proc. Natl Acad. Sci..

[64] Wright, S. *Statistical Genetics in Relation to Evolution*. (Paris, Hermann, 1939).

[65] Goldberg A, Günther T, Rosenberg NA, Jakobsson M (2017). Ancient X chromosomes reveal contrasting sex bias in Neolithic and Bronze Age Eurasian migrations. Proc. Natl Acad. Sci..

[66] Heyer E, Chaix R, Pavard S, Austerlitz F (2012). Sex-specific demographic behaviours that shape human genomic variation. Mol. Ecol..

[67] Karmin M (2015). A recent bottleneck of Y chromosome diversity coincides with a global change in culture. Genome Res.

[68] Rasteiro R, Chikhi L (2013). Female and male perspectives on the Neolithic transition in Europe: clues from ancient and modern genetic data. PLoS ONE.

[69] Downey SS, Haas WR, Shennan SJ (2016). European Neolithic societies showed early warning signals of population collapse. Proc. Natl Acad. Sci..

[70] Colledge S, Conolly J, Shennan S (2004). Archaeobotanical Evidence for the Spread of Farming in the Eastern Mediterranean. Curr. Anthropol..

[71] Stordeur, D. & Willcox, G. Indices de culture et d’utilisation des céréales à Jerf el Ahmar. in *De Méditerranée et d’ailleurs… Mélanges offerts à Jean Guilaine.* 693–710 (2009).

[72] Lotka AJ (1932). The growth of mixed populations: two species competing for a common food supply. J. Wash. Acad. Sci..

[73] Volterra V (1928). Variations and fluctuations of the number of individuals in animal species living together. ICES J. Mar. Sci..

[74] Rascovan N (2019). Emergence and Spread of Basal Lineages of Yersinia pestis during the Neolithic Decline. Cell.

[75] World Health Organization. *Facts about plague* (WHO, 2017).

[76] Livi Bacci, M. *La population dans l’histoire de l’Europe*. (Éd. du Seuil, 1999).

[77] Ramsey CB (2017). Methods for summarizing radiocarbon datasets. Radiocarbon.

[78] Reimer PJ (2013). IntCal13 and marine13 radiocarbon age calibration curves 0–50,000 years cal BP. Radiocarbon.

[79] Furholt, M. Mobility and social change: understanding the European neolithic period after the archaeogenetic revolution. *J. Archaeol. Res*. 10.1007/s10814-020-09153-x (2021).

[80] Csilléry K, François O, Blum MG (2012). B. abc: an R package for approximate Bayesian computation (ABC). Methods Ecol. Evol..

[81] Andraszewicz S (2015). An introduction to Bayesian hypothesis testing for management. Res. J. Manag..

[82] Jeffreys, H. *Theory of probability*. (Clarendon Press; Oxford University Press, 1998).

