# Supplementary material for: Spatially explicit paleogenomic simulations support cohabitation with limited admixture between Bronze Age Central European populations
Source: Commun Biol. 2021 Oct 7;4:1163. doi: 10.1038/s42003-021-02670-5 (PMC8497574; doi:10.1038/s42003-021-02670-5)
Supplement: Supplementary file 1 — Supplementary material [file 42003_2021_2670_MOESM1_ESM.pdf]

# Supplementary material 1: Pairwise Bayes factors

**Table S1.1. Pairwise Bayes factors.** Bayes factors computed between all pairs of the 8 scenarios. It should be read as the Bayes factor $BF_{12}$ of each row ($M_1$) against each column ($M_2$).

| | $M_2$ | Competition | | | | No Competition | | | |
|---|---|---|---|---|---|---|---|---|---|
| $M_1$ | | **Control** | **F** | **P** | **F&P** | **Control** | **F** | **P** | **F&P** |
| **Competition** | **Control** | 1.00 | 4.94 | 0.07 | 1.07 | 0.04 | 0.03 | 0.04 | 0.01 |
| | **F** | 0.20 | 1.00 | 0.02 | 0.22 | 0.01 | 0.01 | 0.01 | 0.00 |
| | **P** | 13.43 | 66.37 | 1.00 | 14.43 | 0.50 | 0.38 | 0.47 | 0.18 |
| | **F&P** | 0.93 | 4.60 | 0.07 | 1.00 | 0.03 | 0.03 | 0.03 | 0.01 |
| **No Competition** | **Control** | 26.61 | 131.53 | 1.98 | 28.60 | 1.00 | 0.75 | 0.93 | 0.35 |
| | **F** | 35.30 | 174.51 | 2.63 | 37.95 | 1.33 | 1.00 | 1.24 | 0.47 |
| | **P** | 28.52 | 141.00 | 2.12 | 30.66 | 1.07 | 0.81 | 1.00 | 0.38 |
| | **F&P** | 75.09 | 371.16 | 5.59 | 80.72 | 2.82 | 2.13 | 2.63 | 1.00 |

**Table S1.2. Pairwise Bayes factors.** Bayes factors computed between all pairs of the 4 scenarios without competition and fixed sampling layers. It should be read as the Bayes factor $BF_{12}$ of each row ($M_1$) against each column ($M_2$).

| | $M_2$ | No Competition | | | |
|---|---|---|---|---|---|
| $M_1$ | | **Control** | **F** | **P** | **F&P** |
| **No Competition** | **Control** | 1.00 | 0.74 | 0.76 | 0.23 |
| | **F** | 1.34 | 1.00 | 1.02 | 0.31 |
| | **P** | 1.32 | 0.98 | 1.00 | 0.30 |
| | **F&P** | 4.38 | 3.26 | 3.32 | 1.00 |

# Supplementary material 2: Model choice cross validation

This section presents the confusion matrices for the various model choice analyses. Lines of each table represent the scenario that have generated the pseudo-observed data while columns represent the relative probability estimated for each tested scenario. 100 replicates have been used in all cases.

**Table S2.1. Detailed scenarios of competition.** Confusion matrix of comparison between 8 scenarios (“Control”, “F”, “P”, “F&P” with and without direct competition), without sampling layer optimization.

| **Probability of model** | | **Competition** | | | | **No Competition** | | | |
|---|---|---|---|---|---|---|---|---|---|
| **Data from model** | | **Control** | **F** | **P** | **F&P** | **Control** | **F** | **P** | **F&P** |
| **Competition** | **Control** | 0.331 | 0.199 | 0.111 | 0.266 | 0.023 | 0.021 | 0.027 | 0.022 |
| | **F** | 0.218 | 0.420 | 0.042 | 0.248 | 0.013 | 0.030 | 0.010 | 0.019 |
| | **P** | 0.098 | 0.060 | 0.313 | 0.077 | 0.129 | 0.103 | 0.115 | 0.104 |
| | **F&P** | 0.282 | 0.251 | 0.092 | 0.279 | 0.022 | 0.032 | 0.018 | 0.024 |
| **No Competition** | **Control** | 0.015 | 0.013 | 0.129 | 0.015 | 0.260 | 0.176 | 0.202 | 0.190 |
| | **F** | 0.023 | 0.021 | 0.088 | 0.024 | 0.202 | 0.310 | 0.111 | 0.223 |
| | **P** | 0.014 | 0.006 | 0.127 | 0.012 | 0.221 | 0.109 | 0.322 | 0.189 |
| | **F&P** | 0.017 | 0.015 | 0.100 | 0.017 | 0.197 | 0.208 | 0.205 | 0.241 |

**Table S2.2. Merged scenarios of competition.** Confusion matrix of comparison between the 4 scenarios without competition merged together in one side and the 4 scenarios with direct competition merged together in the other side, without sampling layer optimization.

| **Model** | **Prob. Competition** | **Prob. No Competition** |
|---|---|---|
| **Competition** | 0.831 | 0.169 |
| **No Competition** | 0.179 | 0.821 |

**Table S2.3. Four scenarios with sampling layer optimization.** Confusion matrix of comparison between the 4 scenarios without competition (“Control”, “F”, “P”, “F&P”) with sampling layer optimization.

| | **Probability of scenario** | | | |
|---|---|---|---|---|
| **Data from scenario** | **Control** | **F** | **P** | **F&P** |
| **Control** | 0.391 | 0.209 | 0.202 | 0.198 |
| **F** | 0.203 | 0.485 | 0.083 | 0.229 |
| **P** | 0.187 | 0.069 | 0.531 | 0.214 |
| **F&P** | 0.173 | 0.242 | 0.215 | 0.370 |

**Table S2.4. Two scenarios with sampling layer optimization.** Confusion matrix of comparison between the 2 scenarios without competition (“Control”, “F&P”) with sampling layer optimization.

| **Scenario** | **Prob Control** | **Prob F&P** |
|---|---|---|
| Control | 0.635 | 0.365 |
| F&P | 0.338 | 0.662 |

# Supplementary material 3: Sampling layer optimization

After the first ABC analysis, which consisted in comparing the relative probability of 8 scenarios where each sample could be sampled either in the pastoralists or in the farmers layer, we used the ABC analysis to fix some population layer when it was possible (see Material and Method) to optimize further estimation by maximizing the exploration of the parameter space. Here, we show that fixing the layer of four populations (those for which the results were unambiguous) improves the estimation.

**Table S3.1. Comparison of the F&P scenario with and without sampling layer optimization.** Relative probabilities and GOF p-values of the F&P scenario without competition (the most probable), with and without sampling layer optimization.

| Model | Probability | GOF p-value |
|---|---|---|
| With sampling layer optimization | 0.970 | 0.800 |
| Without sampling layer optimization | 0.030 | 0.690 |

**Figure S3.1. Goodness-of-fit PCA.** The envelope represents 90% of the simulated data for both scenarios “control” and “F&P” with and without sampling layer optimization. The cross represents the observed data and is always inside the envelope. If the cross was outside the envelope, it would indicate a poor fit of the model.

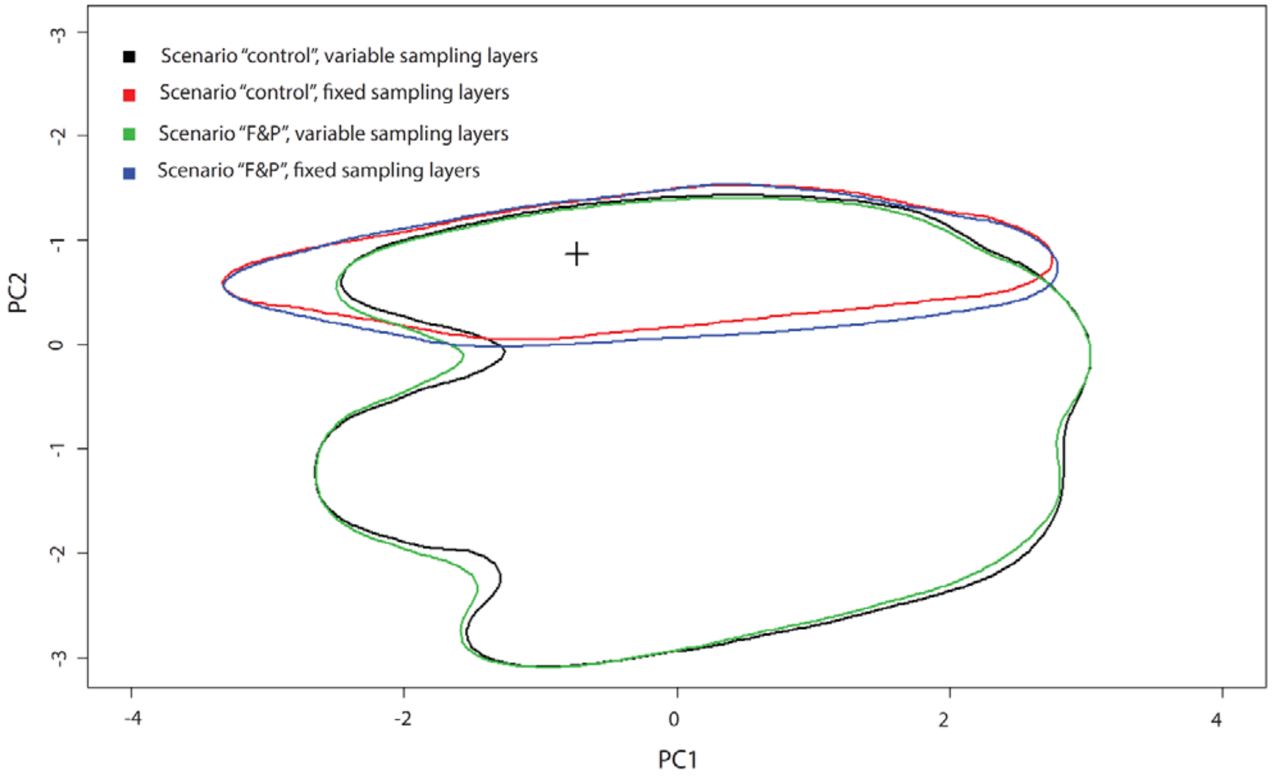

**Table S3.2.** Estimation of the sampling layer based on scenario "F&P" without competition. The R "abc" package was used with loclinear method and tolerance 0.05. The mention "F or P" is returned when HDI99 lower value is lower than 0.5 and HDI99 upper value is greater than 0.5, meaning that the ABC estimation procedure does not allow to fix one layer. When both lower and upper HDI99 are lower than 0.5, value "F" (for farmers) is returned, while when both are greater than 0.5, value "P" (for pastoralists) is returned. The population Esperstedt_MN was not part of this layer optimization procedure as sampling in the "P" layer was never possible.

| Population | HDI99 lower | HDI99 upper | Retained layer |
|---|---|---|---|
| Esperstedt_MN | NA | NA | F |
| Hungary_Gamba_CA | -0.006 | 0.006 | F |
| Corded_Ware_LN | 0.670 | 1.110 | P |
| Karsdorf_LN | 0.598 | 1.015 | P |
| Alberstedt_LN | 0.076 | 1.192 | F or P |
| Bell_Beaker_LN | 0.209 | 1.009 | F or P |
| Benzigerode_LN | 0.423 | 1.160 | F or P |
| Unetice_EBA | 0.137 | 1.029 | F or P |
| Hungary_Gamba_BA | -0.327 | 0.539 | F or P |
| Halberstadt_LBA | 0.295 | 1.152 | F or P |

## Supplementary material 4: Posterior distribution and cross validation

The next figures (Figures S4.1-12) show the results of the parameter estimation for scenario "F&P" without competition, which is the most likely given the model choice procedure and with four fixed sample layers to optimize the results (see Supplementary material 3). On the left panel of each figure, the parameter estimation is represented as probability densities (prior before demographic filter: dotted line; prior after demographic filter: dashed line; posterior distribution: light grey). On the right panel of each figure, the cross-validation result represents the ability of the ABC analysis to retrieve a parameter value, by considering it as pseudo-observation and performing the ABC analysis. 100 replicates are done using the "abc" R package. Those results have been obtained under the best model (*i.e.* F&P, without competition and with the layer optimization).

**Figure S4.1.** Admixture rate (γ)

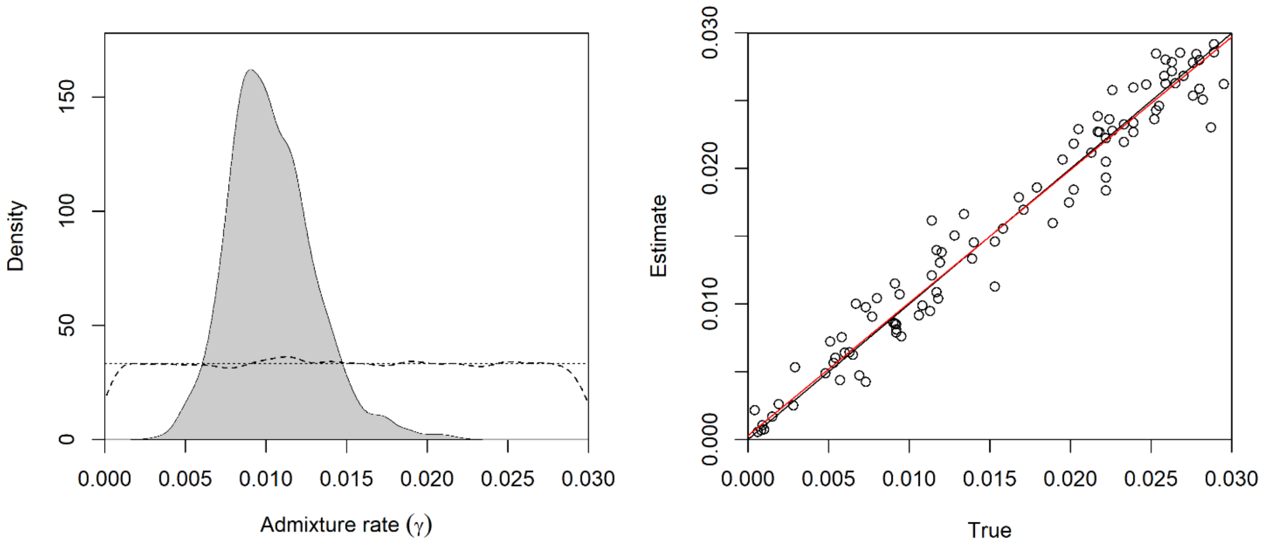


**Figure S4.2.** Long-distance dispersal rate (*LDD*)

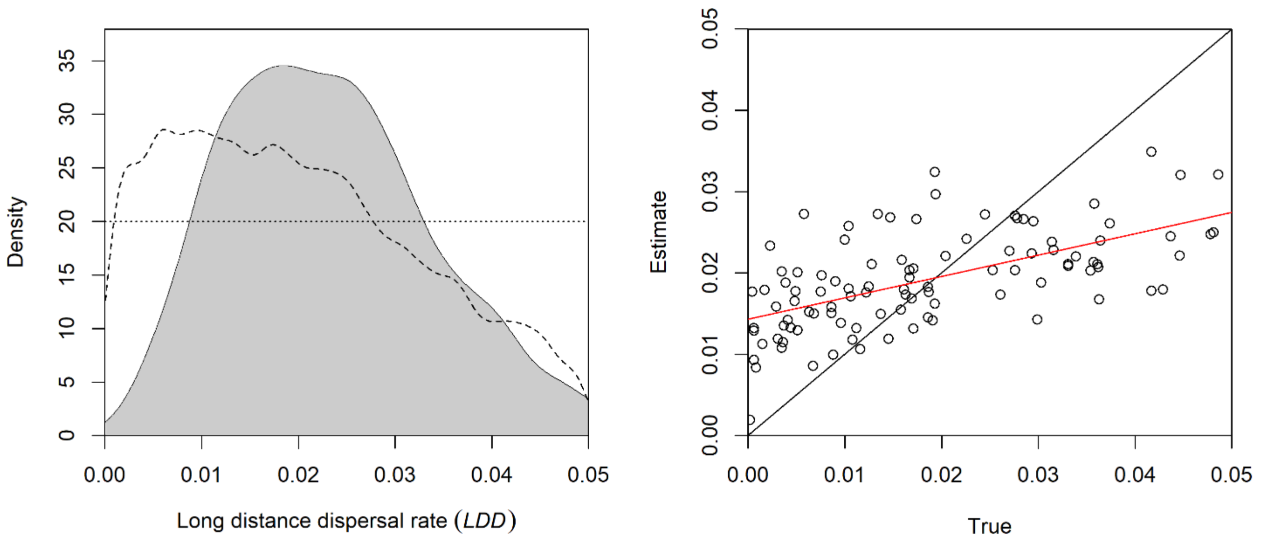

**Figure S4.3.** Population drop rate ($_{S}DD$)

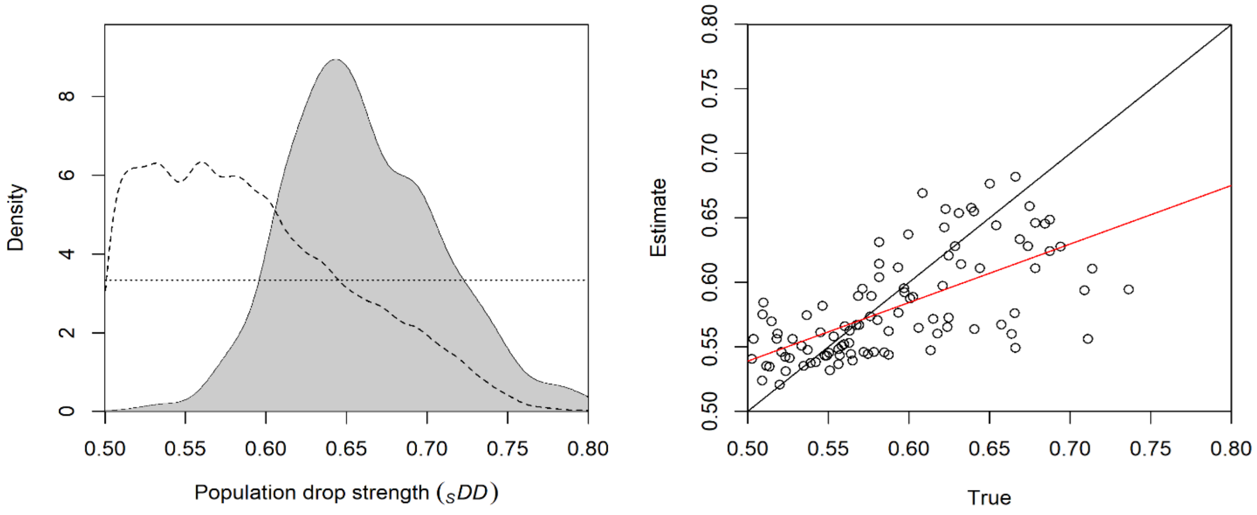


**Figure S4.4.** Absolute number of migrants in the farmer layer ($Nm_F$)

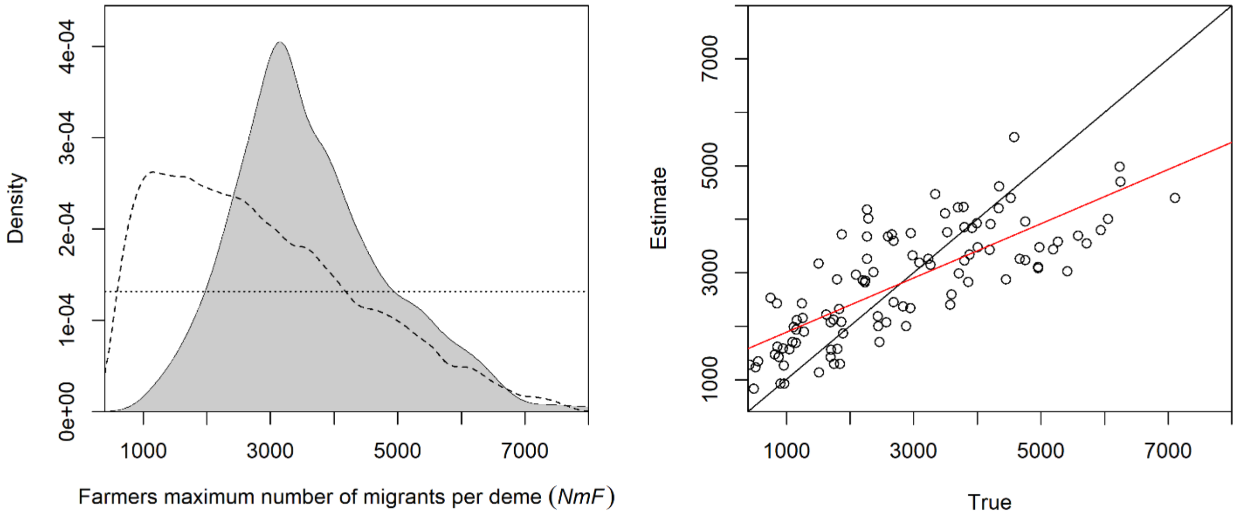


**Figure S4.5.** Absolute number of migrants in the pastoralist layer ($Nm_P$)

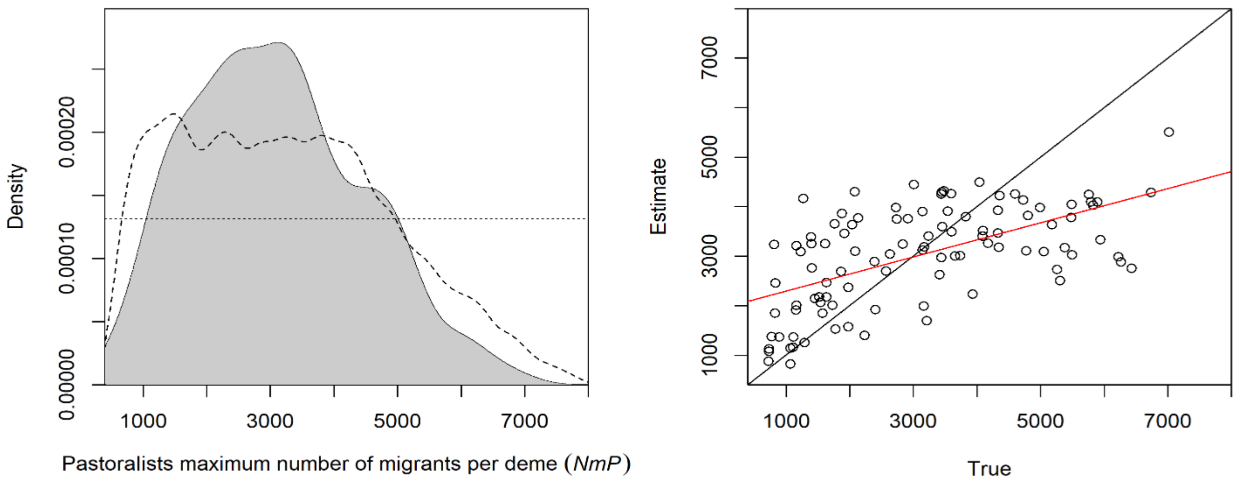

**Figure S4.6.** Carrying capacity farmers ($K_F$)

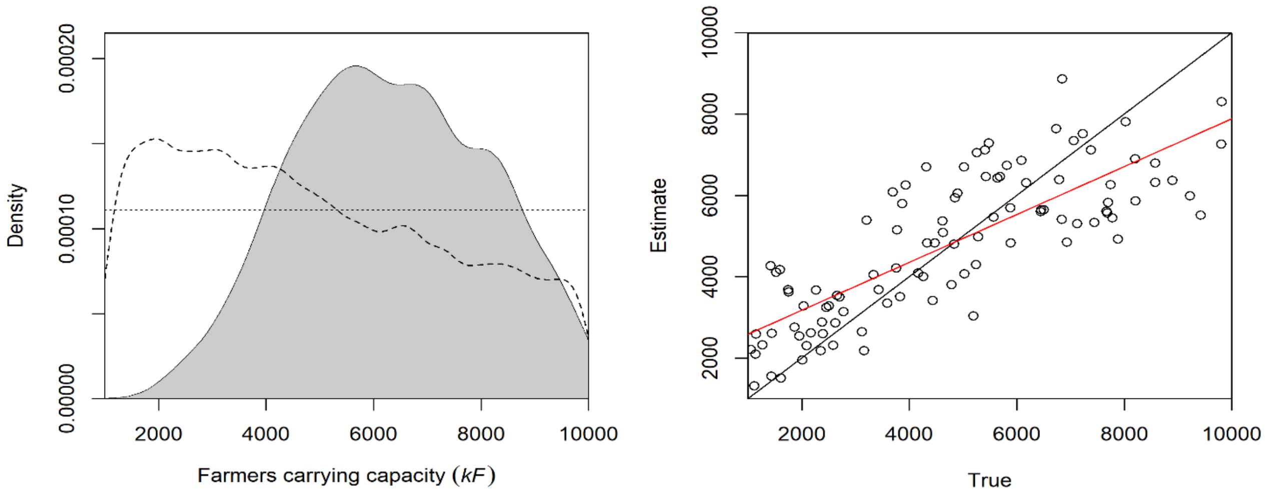


**Figure S4.7.** Carrying capacity pastoralists ($K_P$)

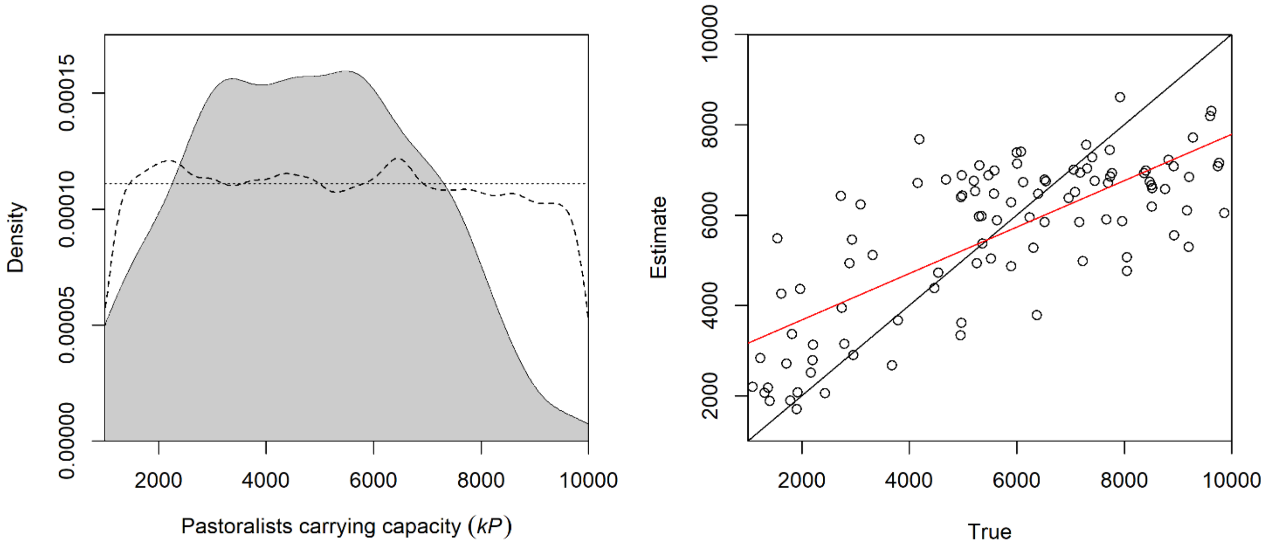


**Figure S4.8.** Growth rate farmers ($r_F$)

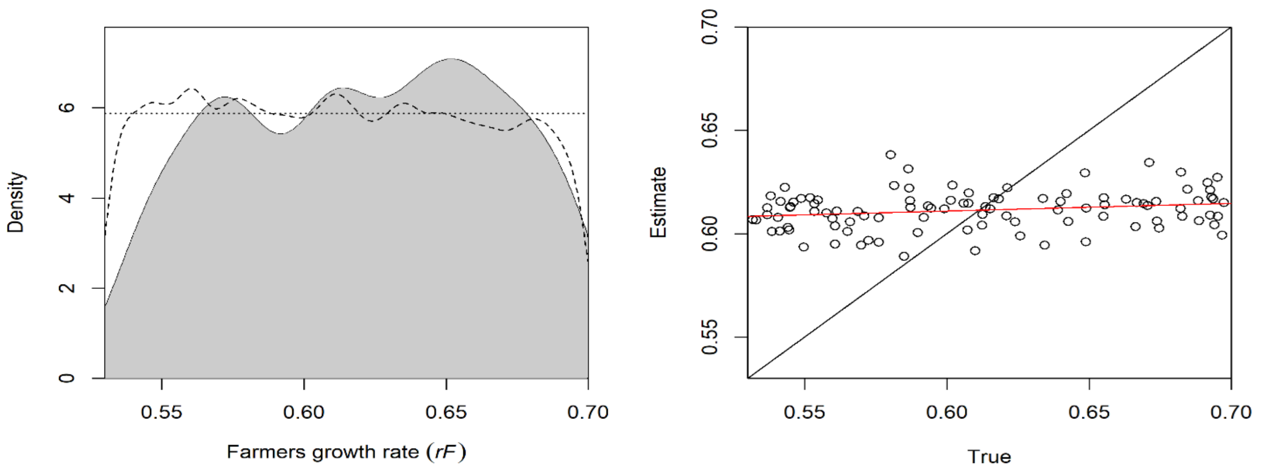

**Figure S4.9.** Growth rate pastoralists ($r_P$)

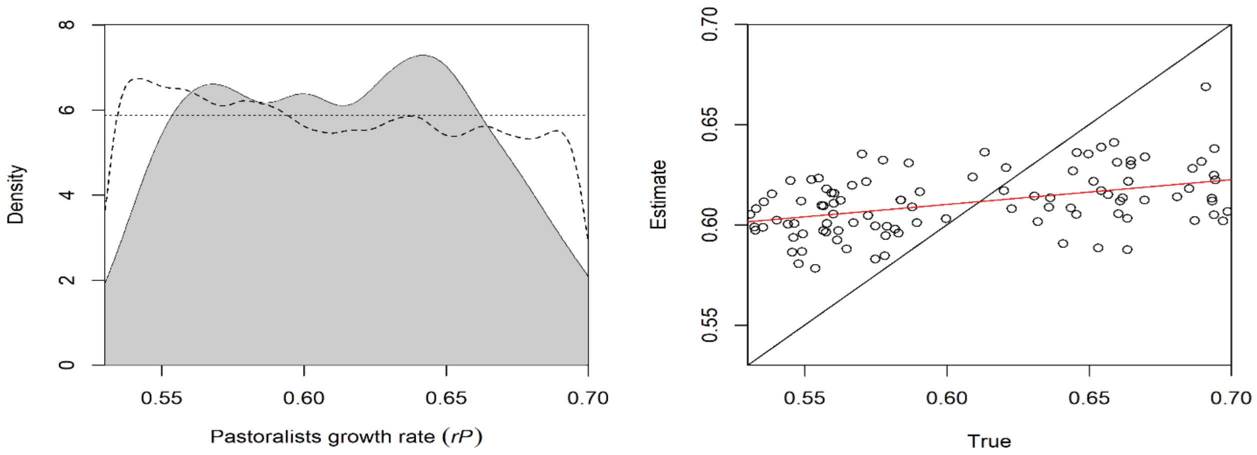


**Figure S4.10.** Population drop ending generation ($_EDD$)

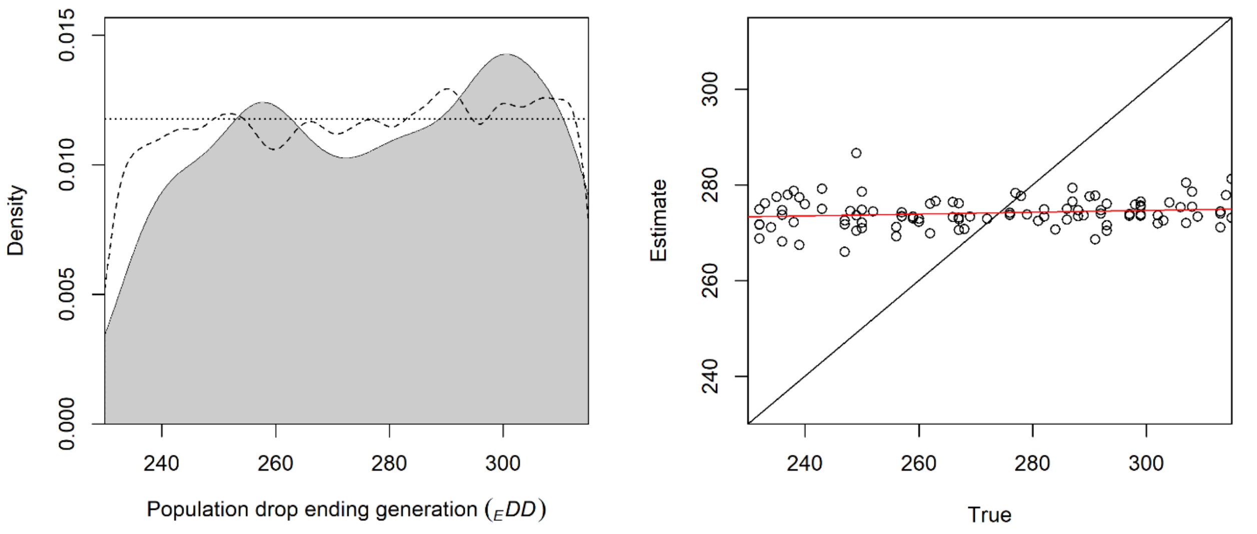


**Figure S4.11.** Migration rate farmers ($m_F$)

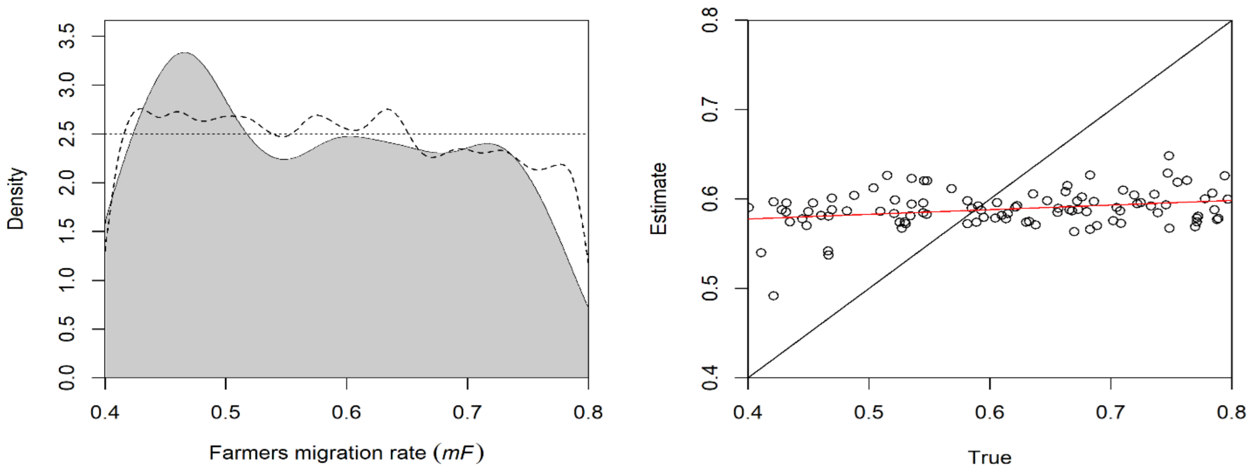

**Figure S4.12.** Migration rate pastoralists ($m_P$)

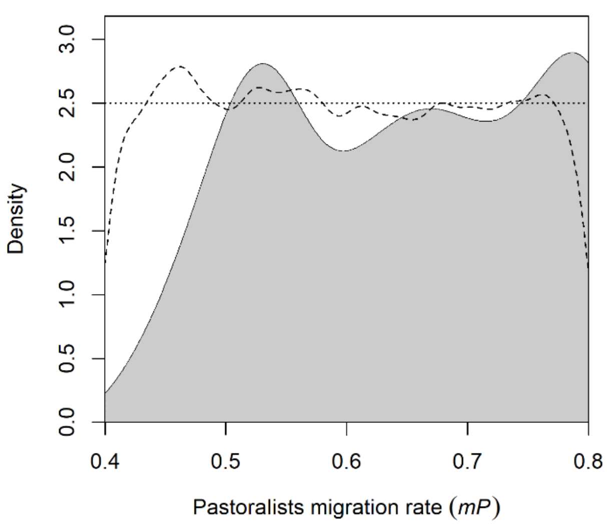


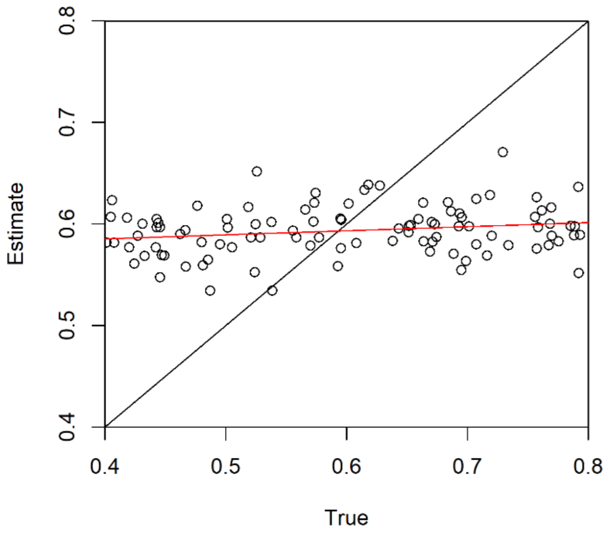

# Supplementary material 5: Long Distance Dispersal

We simulated the expansion of pastoralists to Central Europe under the scenario “control” without competition, with different rates of LDD: 0.0, 0.005, 0.01, 0.014, 0.2, 0.3, 0.4, 0.5. In that situation, we found that the absence of LDD make it impossible to sample the 6 early populations in the pastoralists layer, while the 7$^{th}$ has less than 25% chance to not be sampled (Figure S5: A). When simulating the scenario “F&P” without competition, we observed the same result in the pastoralist layer, but in addition, we also found that some populations taken in the farmer layer has chances not to be sampled (Figure S5: B). This is because sampling is occurring at the time of the demographic drop in some populations and the number of individuals in that deme is not sufficient for sampling to occur. Those results show that a minimum number of LDD is necessary to reach all the sampling demes in Central Europe at the time where they have been sampled, under the most probable conditions (scenario “F&P” without competition).

**Figure S5.11.** Probability for populations to not be sampled, in the farmer layer (F) as well as in the pastoralists layers (P) with different amount of LDD under scenario “control” (A) and “F&P” (B). Each color refers to a different rate of LDD proportion.

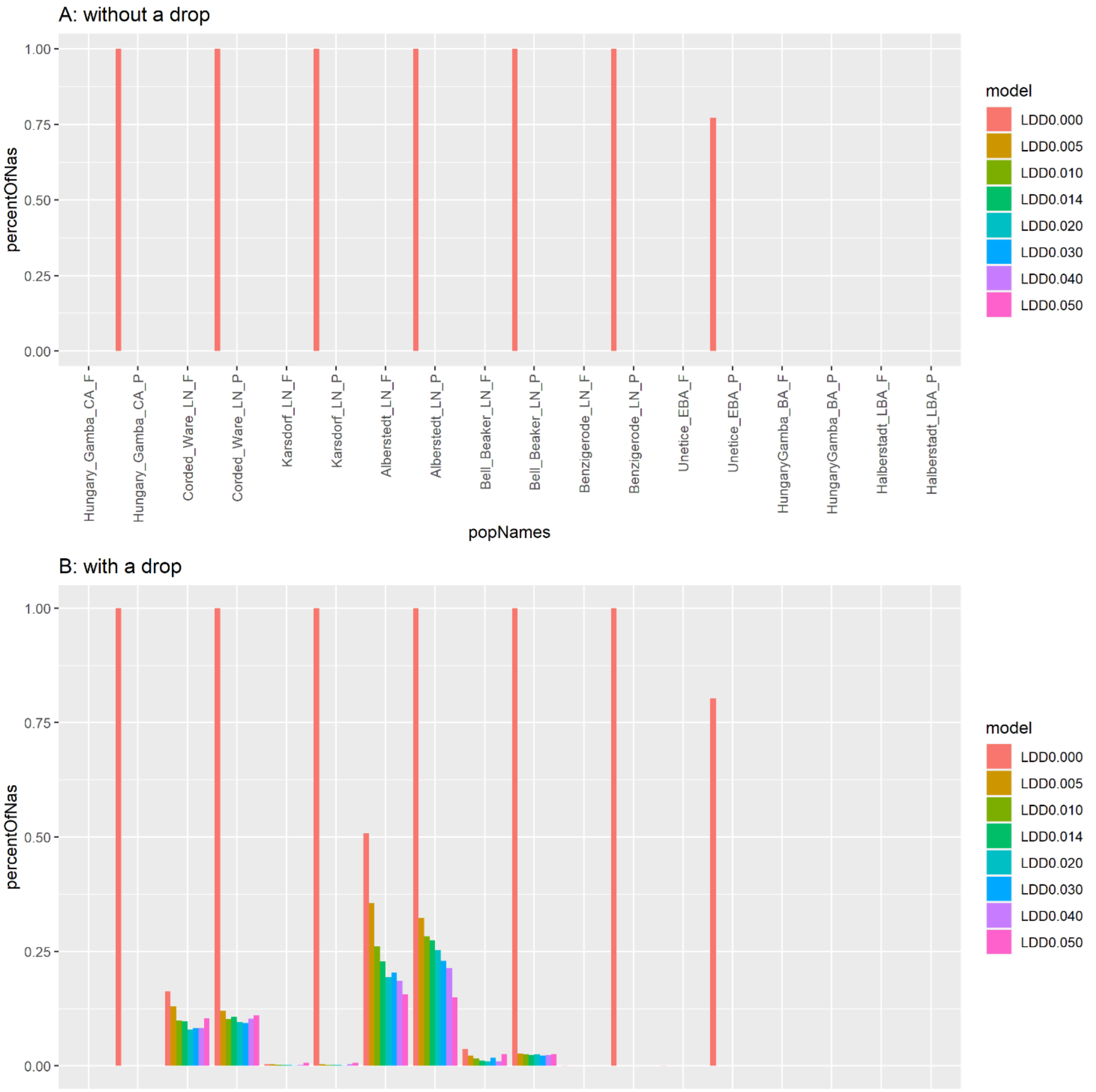

## Supplementary material 6: SPLATCHE3 proportions and f4-ratio

Even if they are expected to be closely linked, the genetic contribution from the pastoralist layer in SPLATCHE3 and the estimated genomic proportions from steppe ancestry associated with the population with Yamnaya culture from Haak *et al.*[1] are not computed in the same way. The former are exact virtual proportions, while the latter are estimated proportions from observed molecular data.

The purpose of this supplementary information is to show the relation between ancestry proportions calculated by the program SPLATCHE3 and the admixture proportions estimated based on f4-ratio statistics in the dataset of Haak *et al.* that we used as observed statistics in our ABC analysis. For this, we thus simulated two different models derived from our study (see below) and for each model we computed the correlation between SPLATCHE3 proportions and f4-ratios computed with the qpAdm function from Admixtools using the R package admixr[2]. Haak *et al*. used an unbiased estimation of those f4-ratios that we are not able to exactly reproduce with SPLATCHE3 because it requires to simulate several worldwide outgroup populations. Nevertheless, we assume that if f4-ratios are correlated to SPLATCHE3 proportions, unbiased f4-ratios will be too as they are supposed to be closer to SPLATCHE3 proportions by definition.

**Material and Methods**

***Models investigated***

We tested two different models to check the relation of SPLATCHE3 proportions and f4-ratios under different parameter context, one is based on the parameter prior distributions (Modelprior) and one is based on the posterior distribution of the model estimated as the best one in our study (Modelposterior).

Modelprior is based on the prior demographic distributions from our simulation framework (Table 3). It uses the middle of the prior range of each demographic parameter as input (including *LDD*, $r_F$, $r_P$, ${}_SDD$, ${}_EDD$, $m_F$, $m_P$). 10 individuals per population are then sampled under the "control" scenario without competition. The admixture rate ranges from 0.006 to 0.03 to explore whether the correlation between SPLATCHE3 proportions and f4-ratio is unbiased across its whole range.

Modelposterior is based on the posterior distribution estimated from our simulations of the scenario "F&P" without competition, which is estimated to be the most likely in our study. Each simulation is made of a random combination of demographic parameters taken in their posterior distributions estimated in our study, ranging from HDI90 lower to upper values (Table 3). The number of individuals per population is identical to the dataset analyzed in our study and taken from Haak *et al.*[1]. Here the idea is to verify that the correlation between SPLATCHE3 proportions and f4-ratio is good in the range of estimated parameter values.

For both models, we tested for the correlation between steppe-ancestry proportions calculated with SPLATCHE3, and admixture proportions estimated with f4-ratios using 1,000 simulated SNP for 100 simulations per model case. The sampling dates are fixed to their average value to avoid more variability than the one already produced by LDD.

***F4-ratio parameters***

Figure S6.1 shows a scheme of the hypothesized phylogenetic tree of the virtual populations under study. The f4-ratio is based on 5 populations, hereafter named X, A, B, C and O.

“X” represents the test populations under study. In the simulations, those populations are sampled in both population layers to show the differences.

“A” represents the farming population from Central Europe having no pastoralists ancestry, from which the f4-ratio statistics, associated to the test populations (X), is compared to. This population is sampled just before the arrival of pastoralists in Central Europe. The sampling date is the same than the real sample "Esperstedt_MN" (generation 193).

“B” represents the ancestral population from Central Europe associated to farmers. This population is sampled just after the moment first pastoralists appear in the simulation: 5,550 BP (generation 178).

“C” represents the ancestral population associated to pastoralists. This population is sampled in Southern Russia just after the moment first pastoralists appear in the simulation: 5,550 BP (generation 178).

“O” is the outgroup population, represented by the first farmers from the Middle East just after the beginning of the simulation: 9,950 BP (generation 2).

The resulting order of the populations that must be taken into account in the f4-statistics is thus: X, A, B, C, O. We can extract the pastoralists ancestry proportion from the resulting f4-ratio using the following equation:

$$\text{pastoralists ancestry proportion} = 1 - \text{f4-ratio}$$

**Figure S6.1.** f4-ratio tree used in both Modelprior and Modelposterior.

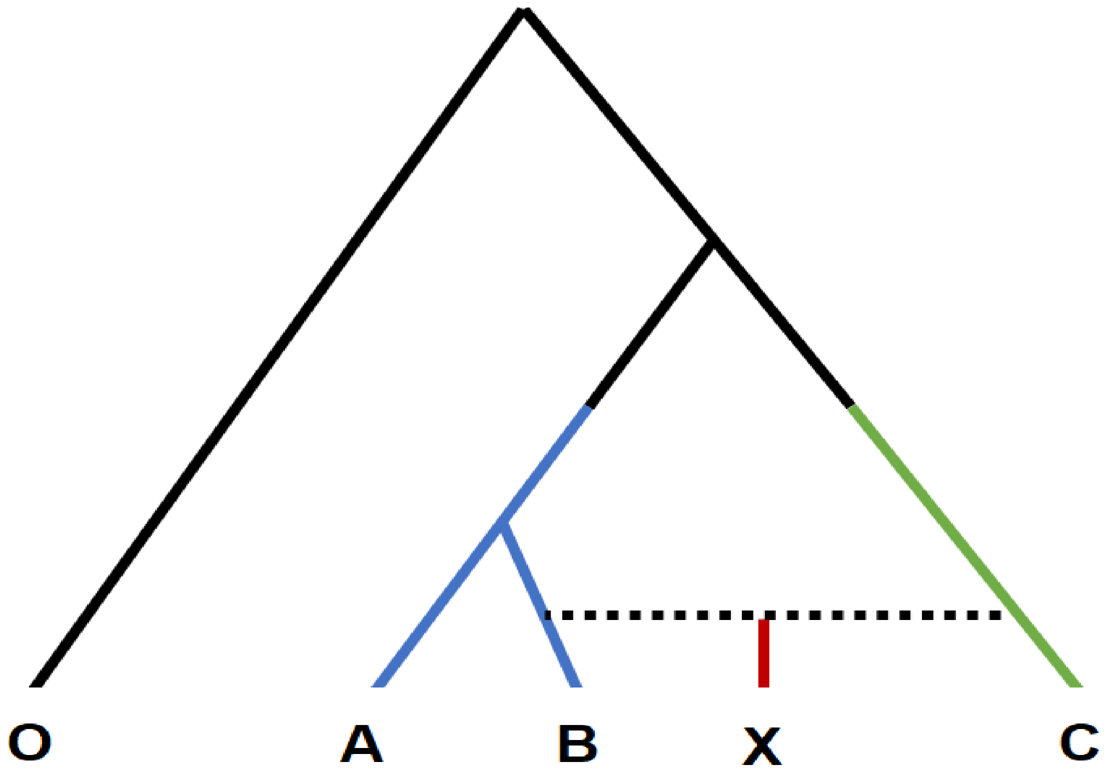

**Figure S6.2.** Locations of population groups used to calculate f4-ratio.

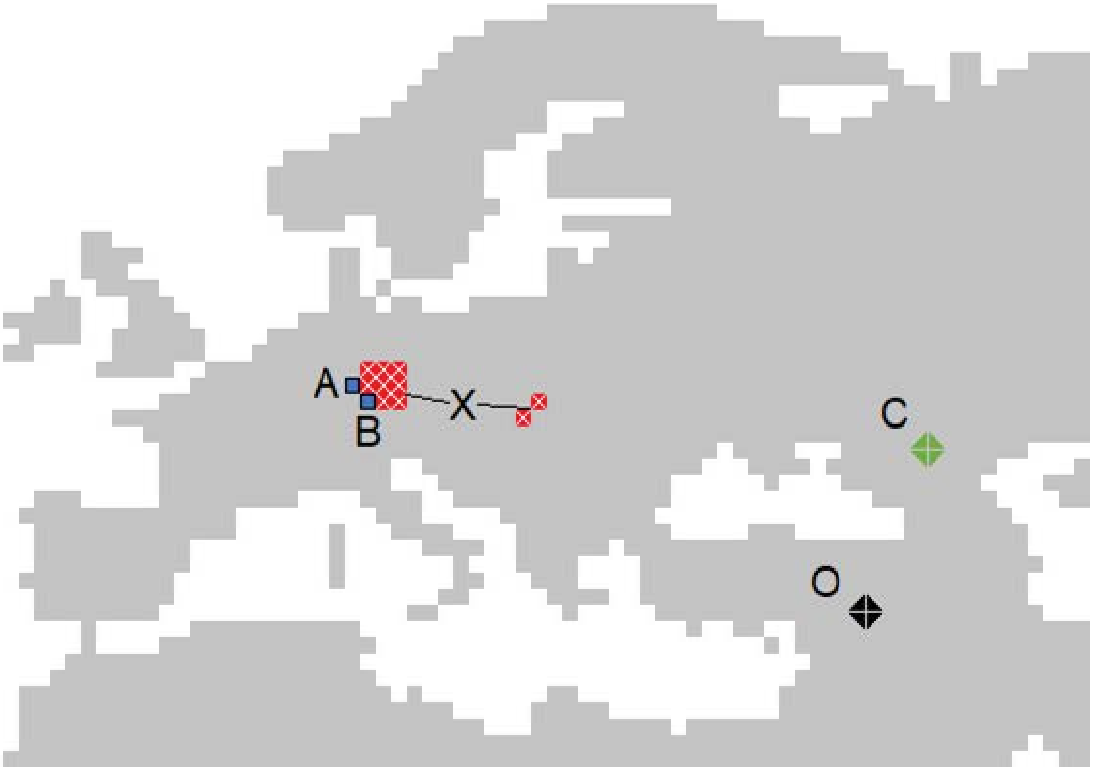


***Pearson correlation***

The Pearson correlation is a parametric test that gives a measure of the association between two continuous variables (here the f4-ratio and the SPLATCHE ancestry). A minimum of 8 values per variable are necessary to get a reliable statistical power. The Pearson correlation is calculated considering all population values (both tested measures are averaged per population).

**Results**

The correlation between SPLATCHE3 and f4-ratio values was tested for Modelprior with different γ values taken within its prior range. In addition, it was also tested for Modelposterior. The mean Pearson correlation is calculated with at least 17 Pearson correlation between f4-ratio and SPLATCHE ancestry (each one made of 1,000 SNP) per γ value (Table S6.1) and with 28 simulations with demographic parameters drawn randomly from the posterior distribution of the best scenario “F&P” (Table S6.1). The number of simulations is not always the same due to demographic filtering (i.e. simulations for which some population samples were not possible). The results show that the correlation decreases linearly with increasing γ (Figure S6.3) and is higher with a larger sample size (Modelprior against Modelposterior).

Moreover, the Figure S6.4. shows two examples of correlation computed sample by sample between SPLATCHE3 proportions and f4-ratio computed under Modelprior, for a low (0.006) and high (0.030) admixture rate (γ) taken within its prior range. Figure S6.5 shows one example of results given by a combination of input parameters for SPLATCHE3 taken from their estimated posterior distribution under Modelposterior.

**Table S6.1.** Pearson correlation in function of the admixture rate γ. SD: Standard Deviation.N: Quantity.

| Model | Admixture rate | Pearson correlation | SD | N simulations | N genomes |
|---|---|---|---|---|---|
| Modelprior | 0.030 | 0.814 | 0.112 | 17 | 10 per population |
| Modelprior | 0.024 | 0.822 | 0.113 | 24 | 10 per population |
| Modelprior | 0.018 | 0.852 | 0.107 | 21 | 10 per population |
| Modelprior | 0.012 | 0.872 | 0.089 | 27 | 10 per population |
| Modelprior | 0.006 | 0.921 | 0.081 | 25 | 10 per population |
| Modelposterior | 0.006 - 0.016 | 0.755 | 0.152 | 28 | Following real data (ranging from 1 to 10) |

**Figure S6.3.** Graph of Pearson correlation in function of γ.

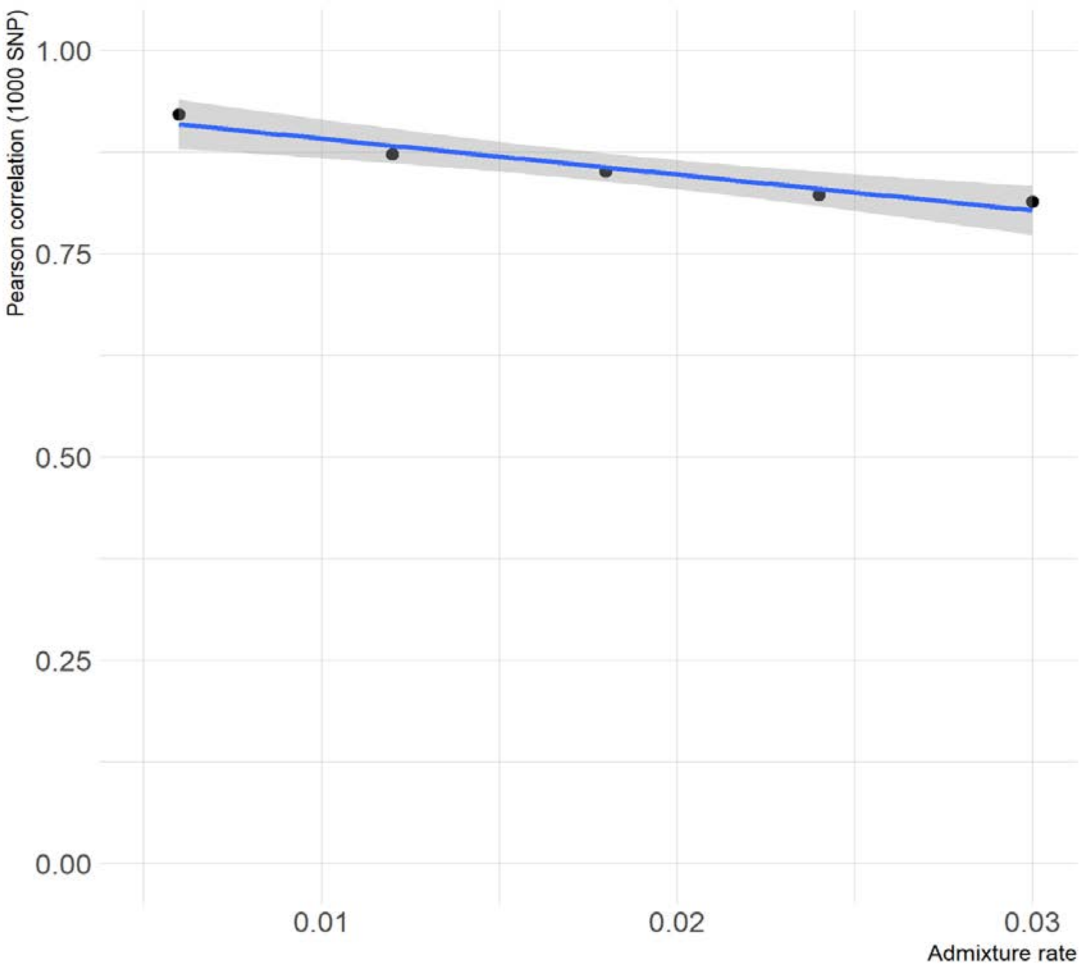

**Figure S6.4.** f4-ratio (red, mean and standard error) and SPLATCHE3 steppe-ancestry proportions (blue, boxplot) based on 1,000 SNP simulated with lower (0.006, left) and higher (0.03, right) admixture rate, with the model based on parameter prior distributions of demographic parameters (Modelprior). “F” denotes a population sampled in the farmers layer, while “P” denotes a population sampled in the pastoralists layer. Ancestry refers to the steppe pastoralist ancestry. The sample Esperstedt_MN can only be taken in the farmer layer.

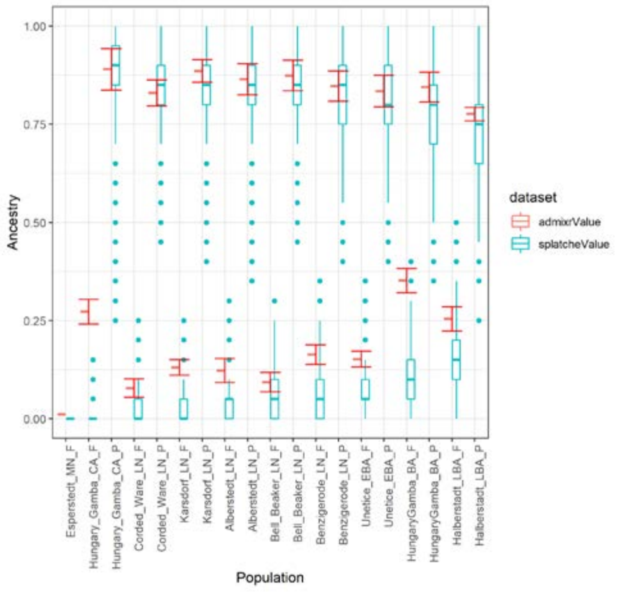


γ = 0.006

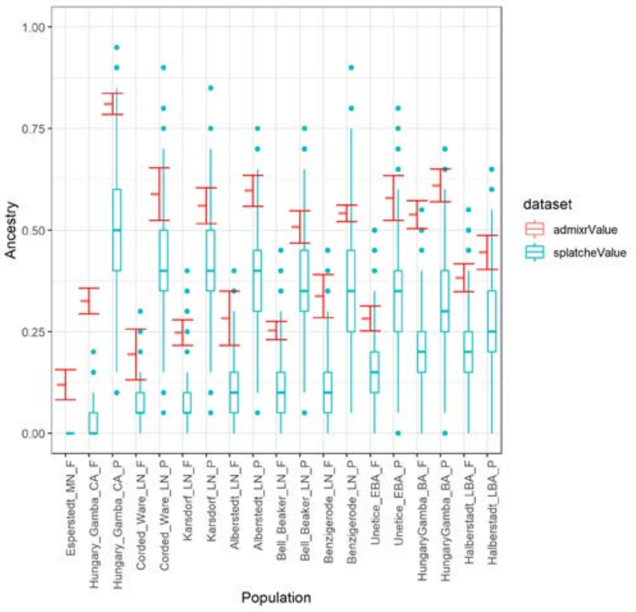


γ = 0.03

**Figure S6.5.** Random example to illustrate f4-ratio (red, mean and standard error) and SPLATCHE3 steppe-ancestry proportions (blue, boxplot) based on 1,000 SNP simulated with the model based on parameter values taken randomly from the posterior distributions of demographic parameters estimated under the most likely model (Modelposterior). “F” denotes a population sampled in the farmers layer, while “P” denotes a population sampled in the pastoralists layer. Ancestry refers to the steppe pastoralist ancestry.

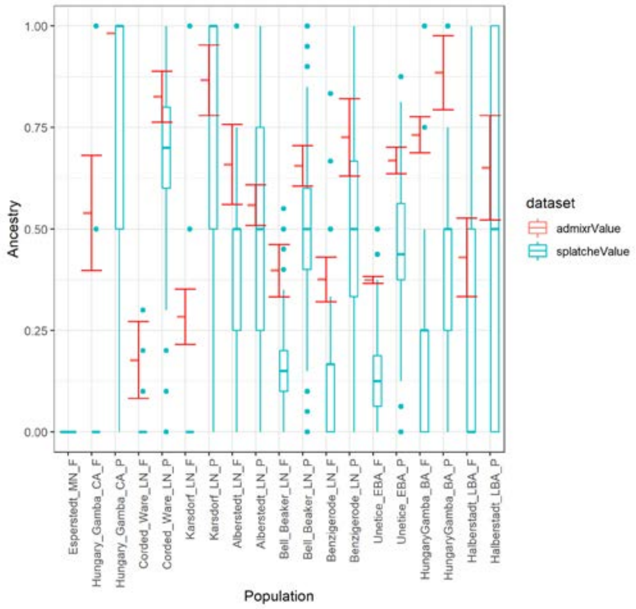

## Discussion

Unbiased admixture proportions estimated by Haak *et al*.[1] and used as observed data in our study are expected to be equivalent to SPLATCHE3 proportions. Our results show that there is indeed a significant correlation between both values (> 75% in all explored cases) and that they can thus be compared.

We observed that the Pearson correlation is higher with lower admixture rate γ than with larger one (Figure S6.3). The γ posterior distribution that we estimated using the ABC analysis is estimated to 1%, corresponding to an expected Pearson correlation between SPLATCHE3 and f4-ratios of about 89% (Table S6.1 and Figure S6.3).

The correlation is better with Modelprior compared to Modelposterior because of the smaller variance in SPLATCHE3 proportions in the former due to larger sample size (10 genomes per sample instead of 1 to 10, see Table 1). Indeed, the variance of SPLATCHE3 proportions is much larger than the variance of f4-ratios due to the stochastic nature of the simulations, which is the reason why 100,000 simulations are used per scenario for the ABC analyses in our study.

As shown by Figure S6.4 and Figure S6.5, the f4-ratio tends to give admixture proportions slightly larger than the steppe-ancestry proportion obtained with SPLATCHE3. This bias increases with the values of γ (higher with larger γ) and decreases with the sample size.

Haak *et al*. improved their estimate of admixture proportion using 15 outgroup populations from different parts of the world, leading to “unbiased” estimates. Due to the complexity of simulating those outgroup populations, we did not reproduce their method exactly. Instead, we compared the steppe-ancestry proportions calculated with SPLATCHE3 to simple estimates of admixture proportions calculated with f4-ratio[3], on which the estimate of Haak *et al.* is based, and we assume that simple f4-ratio and “unbiased” estimates based on those ratios are linked. In theory, “unbiased” population admixture based on f4-ratio should be closer to SPLATCHE3 steppe-ancestry proportion than simple f4-ratio.

Our comparison shows that if there is a bias, it should be in the direction of a slight underestimation of SPLATCHE3 steppe-ancestry proportions compare to classical f4-ratio. In any case, this bias should be comparable for all simulated scenario as they explore identical ranges of parameter values, so giving confidence to the model comparison. The parameter estimation could be slightly biased in favour of parameter values that increases the amount of pastoralist steppe-ancestry proportions in the sample data to compensate for the slight values increasing the proportion of pastoralist in SPLATCHE3. It means potentially a slight overestimation of the admixture rate and of the difference of carrying capacity in favor of the farmers over the hunter-gatherers and a slight underestimation of the amount of long-distance dispersal, which tend to limit genetic introgression from the local population to the invasive population.

# Supplementary material 7: Representativeness of the data analysed

To check the robustness of the paleogenomic pattern investigated and the representativeness of the data analysed in relation to a larger dataset available from various publications, we retrieved from the AADR database (Allen Ancient DNA Resource, version 20 January 2021, https://reich.hms.harvard.edu/allen-ancient-dna-resource-aadr-downloadable-genotypes-present-day-and-ancient-dna-data), all indexes (palaeogenomes) corresponding roughly to the period and area of interest, meaning from Central Europe between latitudes 46° to 53° and longitudes 8° to 22°, dated to the period from 5,300 BP to 2,800 BP (using the "mean BP" value).

By filtering with a mean coverage ≥ 0.05, we extracted 307 indexes in 105 GroupID (population samples). We filtered the dataset for duplicated, closely related and contaminated genomes by removing: i) indexes with same "Master_ID", keeping the one with the highest number of SNPs, ii) the “Master_ID” whose "GroupID" contains a "brother, sister, father, mother, son, daughter, sibling" content in the “GroupID” name, and iii) indexes for which the “GroupID” contains a "contam" or "dup" (contaminated or duplicated) in the “GroupID” name. There are 267 paleogenomes from 67 populations that remain after this filtering, with 1150639 SNPs (1102826 polymorphic). We then added a date to each population, considered later as “target population”, calculated as the average of the "average BP date" of the genomes comprising the GroupID.

We then performed a qpAdm analysis using the program “Admixtools 2” (Maier & Patterson, https://uqrmaie1.github.io/admixtools/index.html), similarly to what was done to estimate the steppe ancestry proportions we are using in our study. To estimate the "steppe ancestry proportions", the qpAdm analysis was done independently on each retained population samples (“GroupID”) from AADR. We choose the “outgroup populations” based on those in Haak et al.[1], namely the WorldFoci15, consisting of 15 global populations. However, the Kharia and Onge populations are not part of the AADR database and the Eskimo population is present as three sub-populations 'Eskimo_Chaplin.DG', 'Eskimo_Sireniki.DG', 'Eskimo_Naukan.DG' in AADR. So, we still have 15 outgroup populations, but slightly different from those used by Haak et al.[1]. We used the following outgroup list: 'Ami.DG', 'Biaka.DG', 'Bougainville.DG', 'Chukchi.DG', 'Eskimo_Chaplin.DG', 'Eskimo_Sireniki.DG', 'Eskimo_Naukan.DG', 'Han.DG', 'Ju_hoan_North.DG', 'Karitiana.DG', 'Mbuti.DG', 'Papuan.DG', 'She.DG', 'Ulchi.DG', 'Yoruba.DG'. The representative genomes of the test populations were chosen as GroupIDs the most similar to those from Haak et al.[1] with “Russia_Samara_EBA_Yamnaya”, “Germany_EN_LBK_Stuttgart_published.DG”. Two samples lead to slightly negative values, but all others have steppes ancestry values between 0.0 and 1.0. The resulting table is called thereafter "AADR" and is displayed in Figure S7.1 below.

We finally compared the datasets analysed in our study (34 paleogenomes in 10 populations, thereafter called “Haak et al 2015”) with the filtered “AADR” dataset of 267 genomes from 67 populations. We divided the period of interest in 5 equal intervals of 500 years (the maximum time step to have at least one population sample from each dataset in each interval, see Figure S7.1). We then calculated the correlation between the average steppe ancestry computed in the two datasets at each time interval. We computed a one-tail Pearson correlation test by using the R package pwr version 1.3, R version 4.0.5. We found a strong positive correlation between the two datasets, statistically significant at the 5% threshold ($r = 0.826$, P value = 0.043). Note that 24 paleogenomes are common to both datasets.

Our analysis shows that the dataset analysed in our study (34 paleogenomes) is robust to the current state of paleogenomic data revealing a general pattern of a sudden increase in steppe ancestry in Central Europe during the Bronze Age.

**Figure S7.1:** Steppe ancestry proportions estimated for 10 population samples (34 genomes) retrieved from the study of Haak et al. (Nature 2015) in blue and estimated with qpAdm from the dataset AADR for 67 populations (267 genomes) in red. Note that reference genomes were not all identical for the analysis of both datasets. 24 genomes are common to both datasets. Vertical lines denote the five-time steps of 500 years considered to compute the correlation between them.

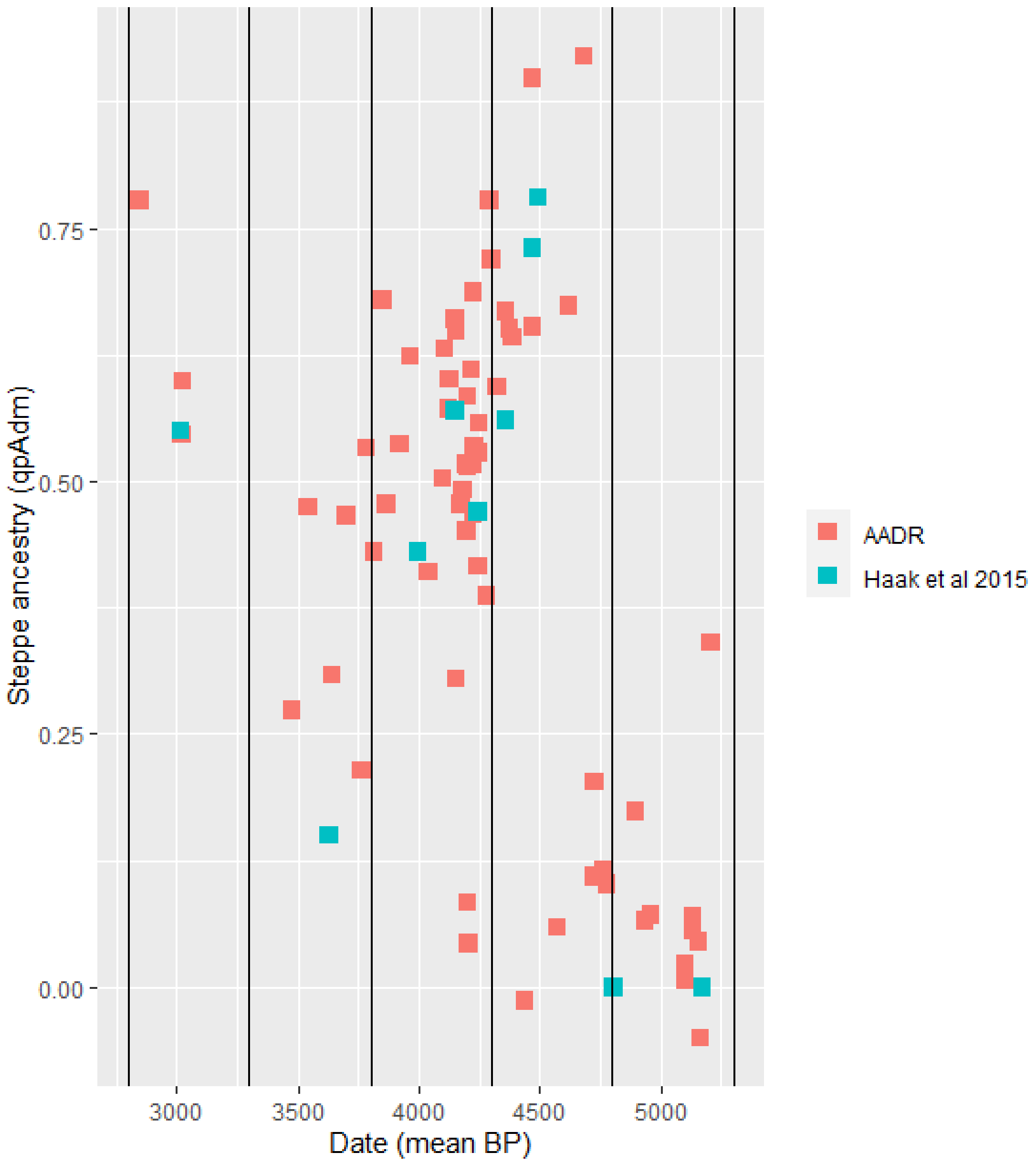

# Supplementary material 8: Conversion of LDD parameters

In the current study, we based the prior values of the long-distance dispersal (LDD) parameters on those estimated by Alves *et al.*[4], but we needed to convert some of them due to the difference in deme size. Indeed, here we used demes of 100*100km while Alves *et al.* used demes of 150*150km. Below are the different LDD parameters in SPLATCHE3:

- $\alpha$: "*Gamma Distribution* shape parameter mode".
- $\mu$: "Averaged number of demes travelled by LDD migrants mode".
- β: "Gamma Scale parameter" corresponds to the inverse of the scale parameter $\theta$ ($\beta$=1/$\theta$).
- $\mu Km$: average distance of LDD in *km*.
- $\sigma Km$: standard deviation of the LDD distance in *km*.
- *ModeKm*: most probable LDD distance in *km*.
- *dist*: length of a deme in *km*, (here 100, or 150 in [4]).

$\alpha$ and $\mu$ are retrieved directly from Alves *et al.* ($\alpha_{150}$=1.209, $\mu_{150}$=5.357) but the other parameters are calculated based on these those two but adapted to demes of 100*100km.

We use the following formulas to retrieve $\beta$, $\sigma$, $\mu Km$, parameters from $\alpha$ and $\mu$.

- $\alpha_{dist}=\mu_{dist}^2/\sigma_{dist}^2$; $\beta_{dist}=(\mu_{dist}/\sigma_{dist}^2)$; $\mu_{dist}=\alpha_{dist}/\beta_{dist}$; $\sigma_{dist}^2=\alpha_{dist}/\beta_{dist}^2$
- $\mu Km=\mu_{dist}*(dist)$; $\sigma Km=\sigma_{dist}*(dist)$; $ModeKm=Mode_{dist}*(dist)$

We convert the average number of demes of 150*150 *km* into distances in *km*:

- $\mu Km$=803.55; $\sigma Km$=730.8; *ModeKm*=138.91

We convert distances back to number of demes, for demes of 100*100 *km*:

- $\mu_{100}$=8.0355; $\sigma_{100}$=7.3080; $Mode_{100}$=1.3891; $\alpha_{100}$=1.209; $\beta_{100}$=0.15046

After conversion, the *Gamma Distribution* corresponding to demes of *150 km* by *150 km* or *100 km* by *100 km* have identical distribution densities, only the scale changes (Figure S8.1). The number of demes that can be migrated considering the gamma distribution for demes of 100 *km* by 100 *km* has grown by 150%. Thus, new formulas can be retrieved from those results, to facilitate the conversion:

- *distA*: deme length before correction,
- *distB*: deme length after correction
- *param*: parameter to be corrected, being Mode, $\mu$, $\sigma$ or $1/\beta=\theta$. The parameter $\alpha$, that controls the shape of the distribution, is not changed after correction (Figure S8.1).
- $param_{\mathrm{distB}} = param_{\mathrm{distA}} * distA/distB$

**Figure S8.1:** Distribution of the long-distance migration probability (represented with a Gamma density) before and after conversion. The shape is the same, only the range of the number of demes is 1.5 times higher after conversion (due to 1.5 times smaller demes side length).

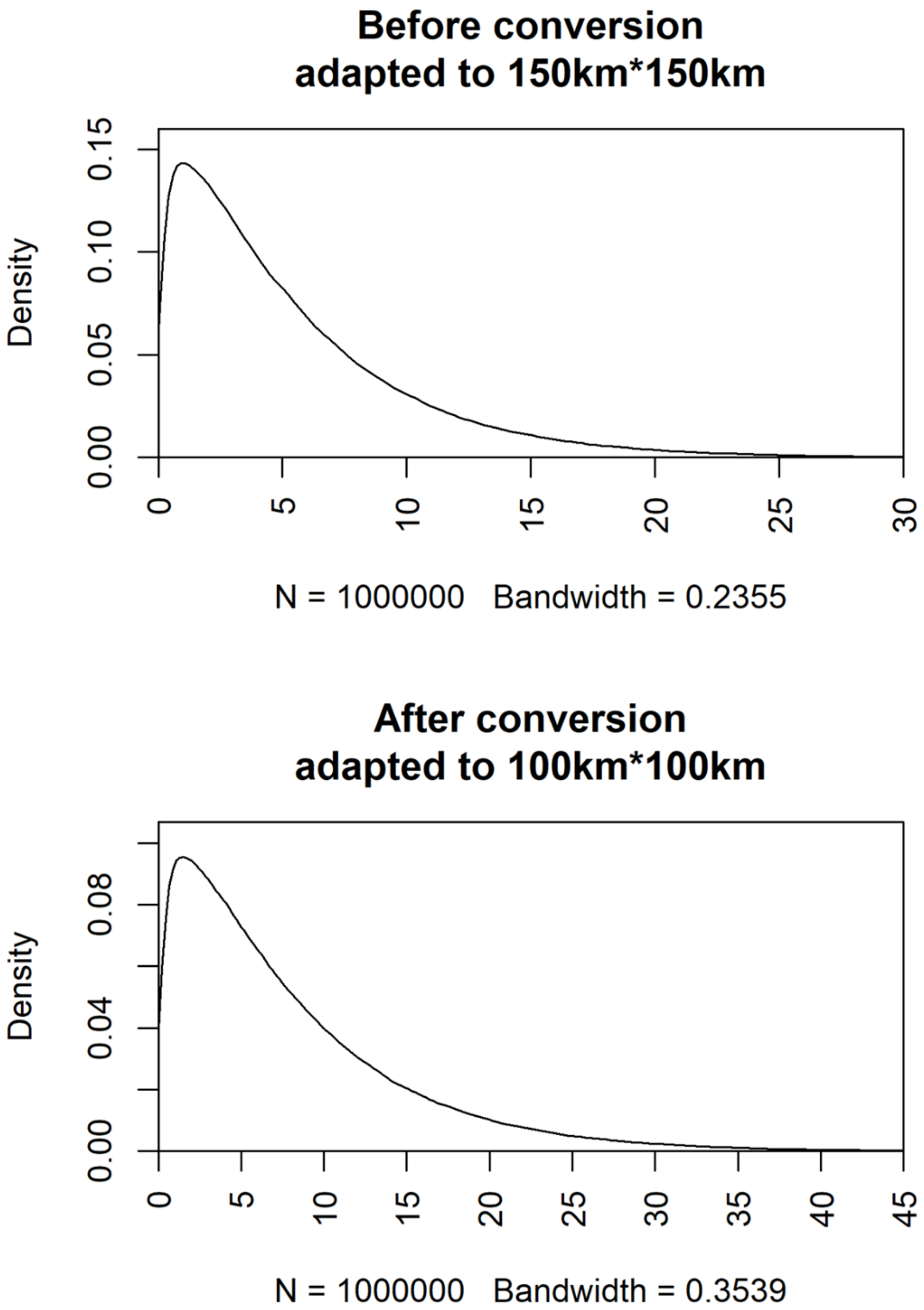

# Supplementary material 9: Dates of the dataset analysed

The dataset analysed in our study has been published in Haak *et al.*[1] Dates given in BP were all converted to calBC with the help of the OxCal 4.3 version project[5], by using the IntCal13 atmospheric curve from[6]. Conversion from calBC to calBP required to add 1950 years.

**Table S9.1.** Archeological dates associated to the 10 populations used in our study.

| individuals | MincalBC | MaxcalBC |
|---|---|---|
| Alberstedt_LN | 2,494 | 2,344 |
| Alberstedt_LN | 2,459 | 2,345 |
| Bell_Beaker_LN | 2,294 | 2,206 |
| Bell_Beaker_LN | 2,497 | 2,436 |
| Bell_Beaker_LN | 2,414 | 2,333 |
| Bell_Beaker_LN | 2,340 | 2,190 |
| Bell_Beaker_LN | 2,290 | 2,130 |
| Bell_Beaker_LN | 2,360 | 2,190 |
| Bell_Beaker_LN | 2,296 | 2,206 |
| Bell_Beaker_LN | 2,345 | 2,198 |
| Bell_Beaker_LN | 3,380 | 2,205 |
| Bell_Beaker_LN | 3,380 | 2,205 |
| Benzigerode_LN | 2,283 | 2,146 |
| Benzigerode_LN | 2,286 | 2,153 |
| Benzigerode_LN | 2,204 | 2,136 |
| Corded_Ware_LN | 2,454 | 2,291 |
| Corded_Ware_LN | 3,960 | 2,117 |
| Corded_Ware_LN | 2,566 | 2,477 |
| Corded_Ware_LN | 2,473 | 2,348 |
| Corded_Ware_LN | 2,454 | 2,291 |
| Esperstedt_MN | 3,360 | 3,086 |
| Halberstadt_LBA | 1,113 | 1,021 |
| HungaryGamba_BA | 2,194 | 2,082 |
| HungaryGamba_BA | 1,266 | 1,181 |
| HungaryGamba_CA | 2,903 | 2,806 |
| Karsdorf_LN | 2,564 | 2,475 |
| Unetice_EBA | 2,131 | 1,979 |
| Unetice_EBA | 1,931 | 1,780 |
| Unetice_EBA | 2,118 | 1,961 |
| Unetice_EBA | 2,199 | 2,064 |
| Unetice_EBA | 2,115 | 1,996 |
| Unetice_EBA | 2,131 | 1,982 |
| Unetice_EBA | 2,131 | 1,979 |
| Unetice_EBA | 2,199 | 2,064 |

## Supplementary material 10: Random choice of 100’000 simulations

This section provides the same analysis as in the main text with a different random subset of 100,000 simulations to show the robustness of model choice and parameter estimation to this subset procedure. The absolute difference between the two subsets is always smaller than 0.04 and the probability order of scenarios is the same (Table 10.1). The estimators for the well estimated parameters (γ, LDD and sDD) is very close with less than 11% relative difference (Table 10.2, while this relative difference increases for the parameters with less precise estimators such as $Nm_F$ and $Nm_P$ (14.5% - 18.4%).

**Table S10.1** Posterior probability for the comparison of 8 scenarios (“Control”, “F”, “P”, and “F&P” with and without direct competition) without sampling layer optimization. To be compared to the Table 2 in the main text. The difference is computed with the posterior probabilities given in the main text using a different random subset.

| Optimization | Model | Scenario | Posterior probability | Difference |
|---|---|---|---|---|
| Variable sampling layers | Competition | Control | 0.007 | 0.001 |
| | | F | 0.001 | 0.000 |
| | | P | 0.111 | 0.037 |
| | | F&P | 0.008 | 0.003 |
| | No Competition | Control | 0.147 | 0.000 |
| | | F | 0.178 | 0.017 |
| | | P | 0.152 | 0.006 |
| | | F&P | 0.395 | 0.020 |

**Table S10.2.** Characteristics of the parameter prior distributions for all the scenarios and characteristics of the posterior distribution for the most likely scenario (“F&P”), with the population layer fixed for four samples. The relative difference between the estimator of this subset ($\hat{\theta}_2$) and the one given in the main text ($\hat{\theta}_1$) is computed as $\frac{|\hat{\theta}_1-\hat{\theta}_2|}{\frac{\hat{\theta}_1+\hat{\theta}_2}{2}}$. To be compared to the Table 3 in the main text.

| | $r_F$ | $r_P$ | γ | *LDD* | *eDD* | *sDD* | $Nm_F$ | $Nm_P$ |
|---|---|---|---|---|---|---|---|---|
| Prior lower | 0.530 | 0.530 | 0.000 | 0.000 | 230 | 0.500 | 400 | 400 |
| Lower 90% HDI | 0.544 | 0.532 | 0.006 | 0.000 | 229 | 0.557 | 2,196 | 496 |
| Weighted Median | 0.621 | 0.616 | 0.011 | 0.020 | 271 | 0.646 | 4,232 | 2,685 |
| Weighted Mean | 0.62 | 0.615 | 0.011 | 0.021 | 269 | 0.649 | 4,395 | 2,774 |
| Weighted Mode | 0.663 | 0.641 | 0.010 | 0.011 | 295 | 0.62 | 3,914 | 2,852 |
| Upper 90% HDI | 0.696 | 0.69 | 0.017 | 0.043 | 307 | 0.749 | 6,472 | 5,106 |
| Prior upper | 0.700 | 0.700 | 0.030 | 0.050 | 315 | 0.800 | 8,000 | 8,000 |
| Relative difference | 0.008 | 0.013 | 0.105 | 0.091 | 0.007 | 0.012 | 0.184 | 0.145 |

# Supplementary material 11: Choice of the number of simulated SNP

To be sure that the number of simulated SNP is high enough to get robust estimates of genomic proportions from source populations (pastoralist of farmer) with SPLATCHE3, we repeated the most probable Scenario “F&P” by simulating 1,000 SNP. Then we compared the resulting genetic proportions of pastoralist with the one obtained with a subset of only 50 SNP (Figure S11.1). It shows that, overall, the steppe-ancestry proportions calculated with 50 SNP give the same results than if it was calculated with 1,000 SNP. We found that the only population showing slightly different steppe-ancestry proportions is the one dated to 226 generations, corresponding to the "Alberstedt" sample. In that case, the sampling occurs right after the demographic drop (starting at 222 generations) so the combination between smaller population sizes and randomness of LDD events increase the variance of ancestry proportions. However, we judged that the gain in computational time due to simulation of only 50 SNP instead of 1,000 SNP (>20x), allowing to better explore the parameter space, worth this slight approximation in rare cases.

**Figure 11.1.** Effect of the number of simulated SNP on resulting genomic proportions outputted by SPLATCHE3. X-axis: number of 25 years generations after 10,000 BP. Y-axis: Amount of steppe-ancestry proportions in population samples. Vertical colored segments relate to the standard errors. Values are averaged over 100 replicates and obtained under scenario “F&P” with the admixture rate fixed to 0.015 and the proportion of LDD fixed to 0.01. All other demographic parameters were fixed to their average values within their prior distribution. The drop occurs at 4,450BP (222 generations, dashed vertical line).

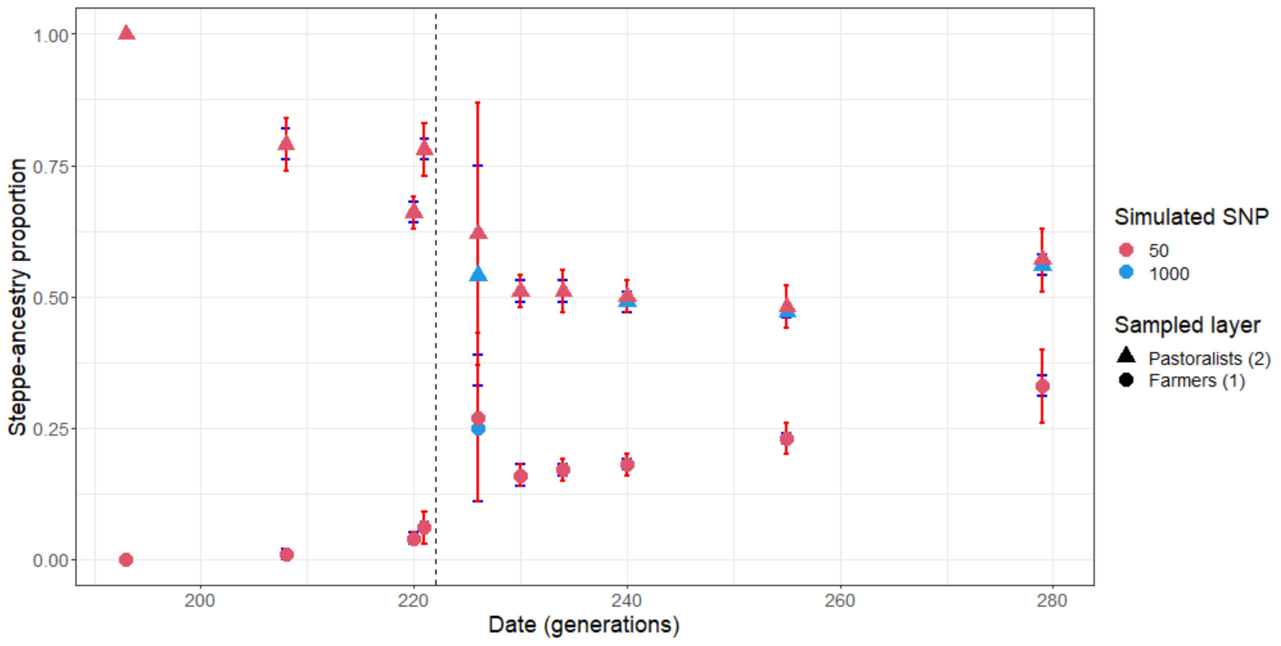

# Supplementary material 12: Choice of tolerance rate for ABC

We also investigated variation of the choice of the tolerance rate for the ABC analyses. We tested different values of tolerance rates between 0.001 (100 retained simulations) and 0.2 (20,000 retained simulations) when comparing the four scenarios without competition (Figure S12.1 and S12.2). We found an instability of the results with low tolerance rate (< 0.01), due to the too low number of retained simulations (Figure S12.1) and a diminution of the goodness of fit measure by the GOF p-values with larger number of tolerance rates (> 0.01). Consequently, we decided to use a tolerance rate = 0.01 (1,000 retained simulations) for all analyses presented in the main text.

**Figure S12.1.** Model choice probability in function of tolerance rate. Scenarios without competition.

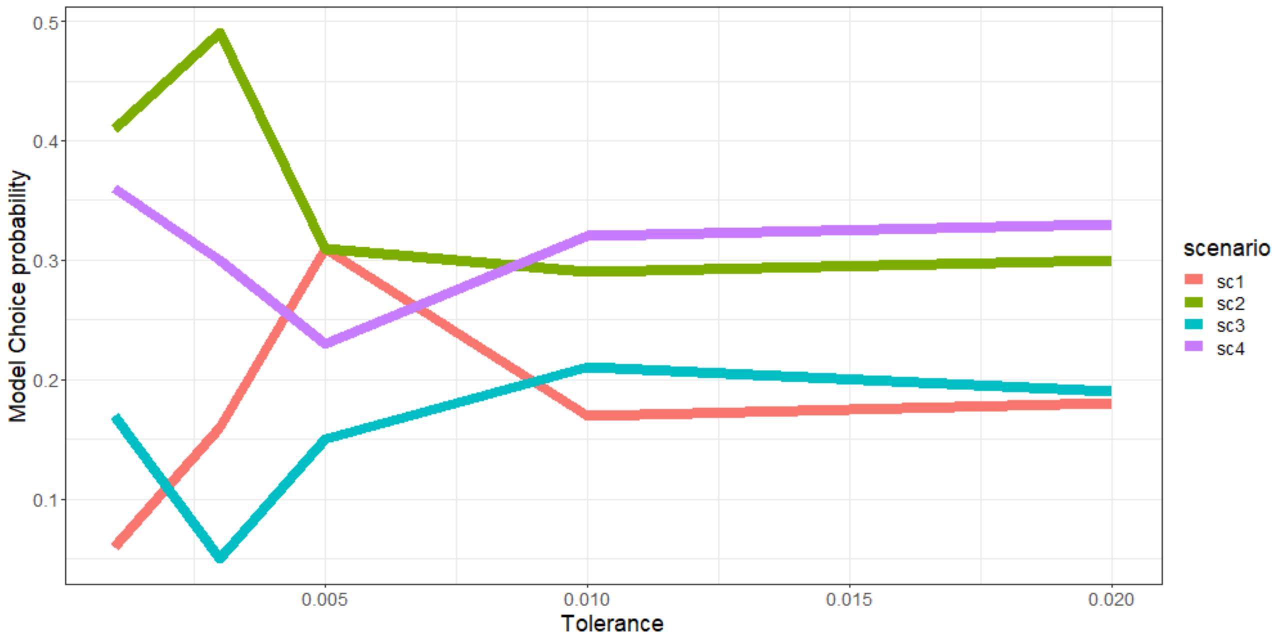


**Figure S12.2.** GOF p-value in function of tolerance rate. Scenarios without competition.

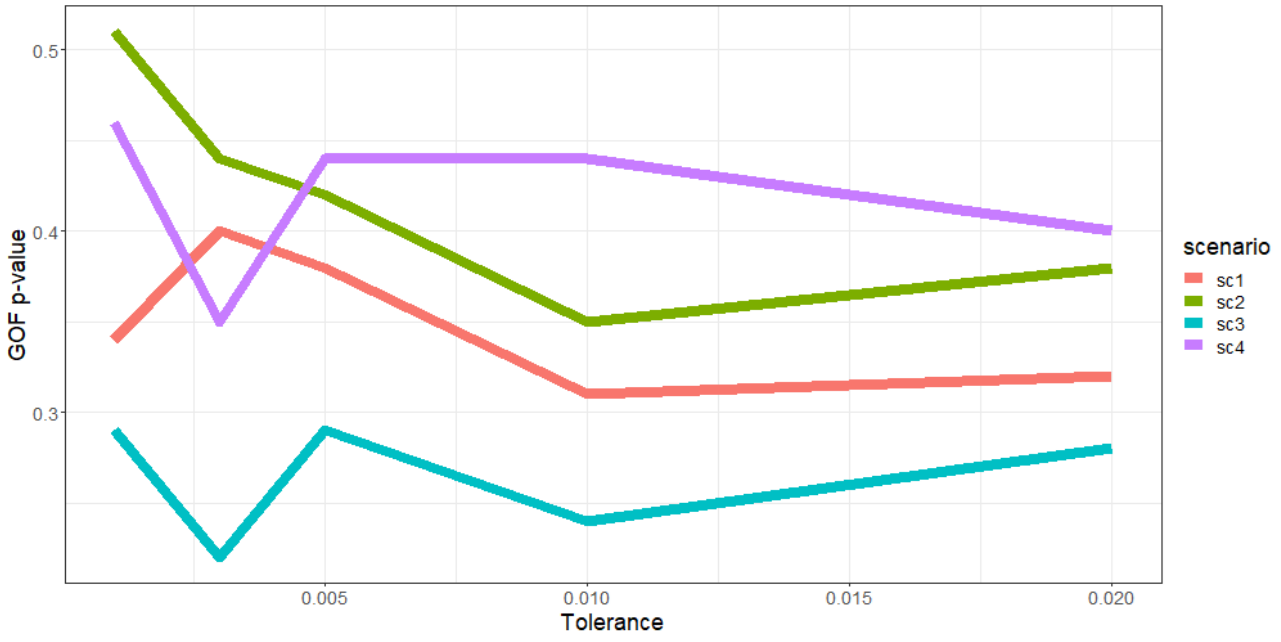

# References


1. Haak, W. *et al.* Massive migration from the steppe was a source for Indo-European languages in Europe. *Nature* **522**, 207–211 (2015).

2. Petr, M., Vernot, B. & Kelso, J. admixr—R package for reproducible analyses using ADMIX-TOOLS. *Bioinformatics* **35**, 3194–3195 (2019).

3. Patterson, N. *et al.* Ancient Admixture in Human History. *Genetics* **192**, 1065–1093 (2012).

4. Alves, I. *et al.* Long-Distance Dispersal Shaped Patterns of Human Genetic Diversity in Eurasia. *Mol. Biol. Evol.* **33**, 946–958 (2016).

5. Ramsey, C. B. Methods for Summarizing Radiocarbon Datasets. *Radiocarbon* **59**, 1809–1833 (2017).

6. Reimer, P. J. *et al.* IntCal13 and Marine13 Radiocarbon Age Calibration Curves 0–50,000 Years cal BP. *Radiocarbon* **55**, 1869–1887 (2013).
