# Supplementary material for: Spatially explicit paleogenomic simulations support cohabitation with limited admixture between Bronze Age Central European populations
Source: Commun Biol. 2021 Oct 7;4:1163. doi: 10.1038/s42003-021-02670-5 (PMC8497574; doi:10.1038/s42003-021-02670-5)
Supplement: Supplementary file 2 — Reporting Summary [file 42003_2021_2670_MOESM2_ESM.pdf]

nature research

Corresponding author(s): Mathias CURRAT

Last updated by author(s): Aug 18, 2021

Reporting Summary

Nature Research wishes to improve the reproducibility of the work that we publish. This form provides structure for consistency and transparency in reporting. For further information on Nature Research policies, see our Editorial Policies and the Editorial Policy Checklist.

Statistics

For all statistical analyses, confirm that the following items are present in the figure legend, table legend, main text, or Methods section.

n/a	Confirmed	
☐	☒	The exact sample size (n) for each experimental group/condition, given as a discrete number and unit of measurement
☐	☒	A statement on whether measurements were taken from distinct samples or whether the same sample was measured repeatedly
☐	☒	The statistical test(s) used AND whether they are one- or two-sided<br>*Only common tests should be described solely by name; describe more complex techniques in the Methods section.*
☒	☐	A description of all covariates tested
☒	☐	A description of any assumptions or corrections, such as tests of normality and adjustment for multiple comparisons
☐	☒	A full description of the statistical parameters including central tendency (e.g. means) or other basic estimates (e.g. regression coefficient) AND variation (e.g. standard deviation) or associated estimates of uncertainty (e.g. confidence intervals)
☒	☐	For null hypothesis testing, the test statistic (e.g. F, t, r) with confidence intervals, effect sizes, degrees of freedom and P value noted<br>*Give P values as exact values whenever suitable.*
☐	☒	For Bayesian analysis, information on the choice of priors and Markov chain Monte Carlo settings
☒	☐	For hierarchical and complex designs, identification of the appropriate level for tests and full reporting of outcomes
☐	☒	Estimates of effect sizes (e.g. Cohen's d, Pearson's r), indicating how they were calculated

Our web collection on statistics for biologists contains articles on many of the points above.

Software and code

Policy information about availability of computer code

Data collection	The observed data on which the analysis is based was retrieved from the publication of Haak et al., Nature 2015 (DOI: 10.1038/nature14317), while the simulated data was produced using the program SPLATCHE3 with settings and executable provided on Zenodo (DOI: 10.5281/zenodo.5205827), as described in the main text.
Data analysis	Packages "admixr" (0.7.1), "abc" (version 2.1). "pwr" (version 1.3) from R (3.6)

For manuscripts utilizing custom algorithms or software that are central to the research but not yet described in published literature, software must be made available to editors and reviewers. We strongly encourage code deposition in a community repository (e.g. GitHub). See the Nature Research guidelines for submitting code & software for further information.

Data

Policy information about availability of data

All manuscripts must include a data availability statement. This statement should provide the following information, where applicable:

- Accession codes, unique identifiers, or web links for publicly available datasets
- A list of figures that have associated raw data
- A description of any restrictions on data availability

10.5281/zenodo.5205827 contains all files required to reproduce the simulations, as well as the raw data to reproduce Figure 2.

Field-specific reporting

Please select the one below that is the best fit for your research. If you are not sure, read the appropriate sections before making your selection.

☐ Life sciences ☐ Behavioural & social sciences ☒ Ecological, evolutionary & environmental sciences

For a reference copy of the document with all sections, see nature.com/documents/nr-reporting-summary-flat.pdf

Ecological, evolutionary & environmental sciences study design

All studies must disclose on these points even when the disclosure is negative.

Study description	Simulation of ancestry proportions from migrating and local populations under various scenarios of human population dynamics during the Bronze Age period in Western Eurasia. Each scenario is made of 100.000 simulations with demographic and migratory parameters drawn randomly in predefined parameter ranges. Using a Bayesian procedure ABC, the most probable scenario and associated parameter values are estimated by comparing formally the statistics simulated under each scenario and combination of parameters to observed data.
Research sample	Samples represent human paleogenomic data from the end of the Neolithic to the beginning of the Bronze Age periods, in Central Europe.
Sampling strategy	Ancestry estimates from 34 published paleogenomes from 10 populations are used. Similar sampled values are simulated.
Data collection	J.R. extracted the real data from Haak et al., Nature 2015 (Figure 3). J.R. produced the simulated data with SPLATCHE3.
Timing and spatial scale	NA
Data exclusions	Samples from the publication of Haak et al., Nature 2015 (Figure 3) that were older than the start of the Yamnaya culture were excluded from the analyses.
Reproducibility	Results coming from another dataset of 100,000 simulations did not change the result interpretation and conclusions. All settings and the executable are provided into a public repository to allow reproducing the simulations and analyses.
Randomization	NA
Blinding	NA

Did the study involve field work? ☐ Yes ☒ No

Reporting for specific materials, systems and methods

We require information from authors about some types of materials, experimental systems and methods used in many studies. Here, indicate whether each material, system or method listed is relevant to your study. If you are not sure if a list item applies to your research, read the appropriate section before selecting a response.

Materials & experimental systems

n/a	Involved in the study	
☒	☐	Antibodies
☒	☐	Eukaryotic cell lines
☒	☐	Palaeontology and archaeology
☒	☐	Animals and other organisms
☒	☐	Human research participants
☒	☐	Clinical data
☒	☐	Dual use research of concern

Methods

n/a	Involved in the study	
☒	☐	ChIP-seq
☒	☐	Flow cytometry
☒	☐	MRI-based neuroimaging
